# A novel porcine model of CLN3 Batten disease recapitulates clinical phenotypes

**DOI:** 10.1242/dmm.050038

**Published:** 2023-08-07

**Authors:** Vicki J. Swier, Katherine A. White, Tyler B. Johnson, Xiaojun Wang, Jimin Han, David A. Pearce, Ruchira Singh, Arlene V. Drack, Wanda Pfeifer, Christopher S. Rogers, Jon J. Brudvig, Jill M. Weimer

**Affiliations:** ^1^ Pediatrics and Rare Diseases Group, Sanford Research, Sioux Falls, SD 57104, USA; ^2^ Precigen Exemplar, Coralville, IA 52241, USA; ^3^ Department of Ophthalmology, Center for Visual Science, University of Rochester Medical Center, Rochester, NY 14642, USA; ^4^ Department of Ophthalmology and Visual Sciences, University of Iowa, Iowa City, IA 52242, USA; ^5^ University of Iowa Institute for Vision Research, Iowa City, IA 52242, USA; ^6^ Department of Pediatrics, Sanford School of Medicine, University of South Dakota, Sioux Falls, SD 57105, USA

**Keywords:** Neuronal ceroid lipofuscinosis, JNCL, Animal disease models, Neuropediatric disease

## Abstract

Mouse models of CLN3 Batten disease, a rare lysosomal storage disorder with no cure, have improved our understanding of CLN3 biology and therapeutics through their ease of use and a consistent display of cellular pathology. However, the translatability of murine models is limited by disparities in anatomy, body size, life span and inconsistent subtle behavior deficits that can be difficult to detect in CLN3 mutant mouse models, thereby limiting their use in preclinical studies. Here, we present a longitudinal characterization of a novel miniswine model of CLN3 disease that recapitulates the most common human pathogenic variant, an exon 7-8 deletion (*CLN3^Δex7/8^*). Progressive pathology and neuron loss is observed in various regions of the *CLN3^Δex7/8^* miniswine brain and retina. Additionally, mutant miniswine present with retinal degeneration and motor abnormalities, similar to deficits seen in humans diagnosed with the disease. Taken together, the *CLN3^Δex7/8^* miniswine model shows consistent and progressive Batten disease pathology, and behavioral impairment mirroring clinical presentation, demonstrating its value in studying the role of CLN3 and safety/efficacy of novel disease-modifying therapeutics.

## Teaser

**Summary:** Characterization of a novel porcine model of CLN3 Batten disease showing behavioral, pathological and ocular deficits as seen in individuals with CLN3, demonstrating its value in studying this disease and the efficacy of various therapeutics.

## INTRODUCTION

Batten disease (also known as neuronal ceroid lipofuscinoses, NCLs) is a family of more than ten different autosomal recessive, pediatric, neurodegenerative disorders that severely reduces quality of life and leads to premature death (Mole and Cotman, 2015). The CLN3 subtype (resulting from mutations in the *CLN3* gene and referred to here as CLN3 disease) is the most common form of Batten disease in the United States and Europe (Wisniewski et al., 1998), and has a juvenile onset of symptoms between 4 and 7 years of life (Hofmann et al., 1999). Symptoms typically initiate with visual impairment (with loss of photoreceptors), followed by cognitive decline, loss of motor function (including impaired balance and shuffling gait), seizures and premature death by the second or third decade (Johnson et al., 2019a; Jalanko and Braulke, 2009; Anderson et al., 2013; Williams et al., 2006; Wright et al., 2020). Pathologically, accumulation of lysosomal storage material, glial activation and neuronal degeneration are key hallmarks of the disease (Cotman et al., 2002). More than 90 different pathogenic variants have been identified in the *CLN3* gene (https://www.ucl.ac.uk/ncl-disease/mutation-and-patient-database/mutation-and-patient-datasheets-human-ncl-genes/cln3), the most common being a 966 bp deletion in exons 7 and 8 (Δex7/8) (Lerner et al., 1995). Of individuals diagnosed with CLN3, 74% were homozygous for this variant and 22% were heterozygous (Munroe et al., 1997; Cotman and Staropoli, 2012).

Multiple mouse models of CLN3 disease have been developed and characterized, including *Cln3^−/−^* knockout models (Weimer et al., 2009; Mitchison et al., 1999; Kovacs et al., 2006; Pontikis et al., 2004; Weimer et al., 2007; Seigel et al., 2002; Weimer et al., 2006; Parviainen et al., 2017), *Cln3^LacZ/LacZ^* knock-in models (Eliason et al., 2007), *Cln3^Δex7/8^* neo knockout (Katz et al., 1999, 2008b) and *Cln3^Δex7/8^* knock-in models with the common human mutation (Cotman et al., 2002; Burkovetskaya et al., 2017; Osorio et al., 2009; Finn et al., 2011). Focusing on the *Cln3^Δex7/8^* knock-in models, these mice develop classic autofluorescent storage material accumulation, reactive deposits of mitochondrial ATP synthase subunit C (SubC), and activation of microglial and astrocytic cells throughout the brain (Cotman et al., 2002; Pontikis et al., 2005; Burkovetskaya et al., 2014). *Cln3^Δex7/8^* mice also demonstrate variable motor declines evident in gait and coordination tests (Cotman et al., 2002; Finn et al., 2011; Osorio et al., 2009; Bosch et al., 2016; Kovacs and Pearce, 2015), and loss of b-wave retinal function as assessed by electroretinography (Volz et al., 2014; Staropoli et al., 2012; Kleine Holthaus et al., 2020; Zhong et al., 2020), which has also been documented in *Cln3^−/−^* mice (Katz et al., 2008b). However, these behavioral phenotypes are subtle and often difficult to recapitulate across laboratories (Centa et al., 2020; Bosch et al., 2016; Finn et al., 2011; Kovacs and Pearce, 2015), and *Cln3^Δex7/8^* mice have a near-normal lifespan (Cotman et al., 2002), indicating that *Cln3* mutations in mouse models lack crucial elements of translatability. Moreover, slight variations in breeding strategies – e.g. of the background strain – behavior testing paradigms, and/or animal husbandry and environmental enrichment can impact consistency in reported outcomes, including biofluid-based biomarkers, in these CLN3 mutant mouse lines (Johnson et al., 2019b; Timm et al., 2018).

To overcome some of the limitations in the murine models of CLN3 disease, we engineered a novel *CLN3^Δex7/8^* miniswine model. Transgenic swine provide a powerful translational tool for modeling human diseases that are poorly recapitulated in smaller animal models (Rogers et al., 2008b; Sieren et al., 2014; Khanna et al., 2019; White et al., 2018; Beraldi et al., 2015; Davis et al., 2014; Park et al., 2015), with their use as a model organism gaining popularity over the past 30 years (Gutierrez et al., 2015). More recently, the US Food and Drug Administration (FDA) approved a domesticated pig line as the first use of a genetically engineered animal for both food and biomedical/therapeutic purposes (https://www.fda.gov/news-events/press-announcements/fda-approves-first-its-kind-intentional-genomic-alteration-line-domestic-pigs-both-human-food). Additionally, swine models are especially useful in neurodegenerative disease modeling due to their gyrencephalic brains, and useful in therapeutic screening as their brains are similar in size to those of humans and, thus, possess a more human-like physiology and pharmacokinetics. In general, large animal models for Batten disease, such as the CLN1 (Eaton et al., 2019), CLN5 (Frugier et al., 2008) and CLN6 (Jolly et al., 1980; Bond et al., 2013) sheep models, as well as the CLN2 dog (Katz et al., 2008a, 2014, 2015, 2005) and CLN2 pig models (Swier et al., 2022), show more clinically relevant symptoms of the disease. Moreover, lifespan of these model animals is longer, allowing the long-term evaluation of disease course, and the effectiveness and safety of therapeutics (Ellinwood and Clay, 2009). Given these advantages, our team developed a novel porcine model of CLN3 disease that replicates the histopathological, behavioral and visual abnormalities experienced by patients with CLN3 Batten disease.

## RESULTS

### CLN3^Δex7/8^ miniswine generation, study design and general observations

Recombinant adeno-associated virus (rAAV)-mediated gene targeting was used to introduce the 966 bp deletion in exons 7 and 8 (Δex7/8) in Yucatan miniswine fetal fibroblasts as previously described (White et al., 2018; Beraldi et al., 2015; Rogers et al., 2008b; Sieren et al., 2014). Briefly, male Yucatan fetal fibroblasts were infected with rAAV carrying a targeting construct designed to replace the endogenous *CLN3* exons 7 and 8 with a neomycin resistance (NeoR) cassette (Fig. S1A), followed by removal of the cassette through Cre recombinase-mediated excision. The resulting *CLN3^+/Δex7/8^* fibroblasts were used as nuclear donors for somatic cell nuclear transfer (SCNT) as previously described (White et al., 2018; Swier et al., 2022). Following SCNT, reconstructed embryos were transferred to recipient miniswine. CLN3-targeted miniswine were born following a 114-day gestation, and heterozygote progenitor miniswine were then bred to expand the colony and generate homozygotes. Successful *CLN3^Δex7/8^* genetic modification was confirmed by using PCR and Southern blots (Fig. S1B,C).

**Fig. 1.**
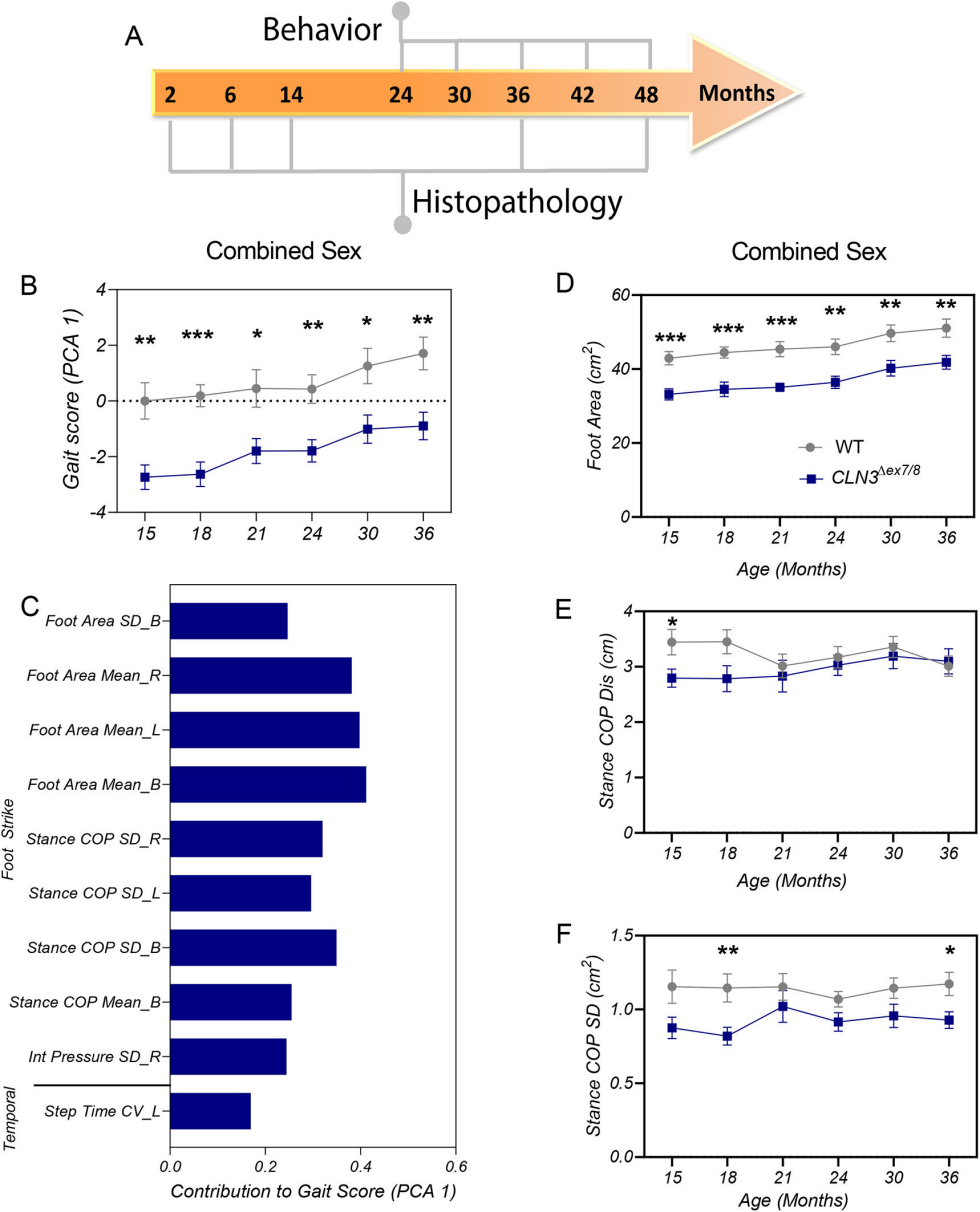
**Study design and gait score.** (A) Study design showing time points for behavior testing and histopathology throughout the study period. (B) Plotted is the PCA gait score for front feet obtained from combined-sex datasets. *CLN3^Δex7/8^* animals have a significantly different gait than wild-type animals at all time points. (C) Description of variables contributing to gait scores for combined-sex datasets. (D) Plotted is the foot area for combined-sex datasets. *CLN3^Δex7/8^* miniswine have a significantly decreased footfall size compared to that of wild-type swine at all time points. (E,F) Stance center-of-pressure (COP) variables for combined-sex datasets. *CLN3^Δex7/8^* miniswine have less variability in stance COP trajectories, indicative of a cautious balancing strategy and more-controlled stance. Mean±s.e.m. Two-way ANOVA with uncorrected Fisher's LSD post-hoc test. *P≤0.05, **P≤0.01, ***P≤0.001. B, both, R; right, L; left; SD, standard deviation; CV, coefficient of variation. Numbers of animals tested are listed in Table S4.

A schematic of the study design is presented in Fig. 1A, with 29 animals monitored up to the age of 36 months and five animals monitored up to the age of 48 months of age. Body condition score was maintained for all animals over the longitudinal study and no seizures were observed. Social and feeding behavior was considered normal for all animals and no obvious walking impairments were observed. No differences in survival were noted during the 24- to 48-month study period, as Yucatan miniswine can live up to 13-15 years (Chieppa et al., 2014).

When examining the weight of all male and female animals during the 24- to 48-month study period, *CLN3^Δex7/8^* miniswine and wild-type pigs had similar weights at all time points (Fig. S2A). When split by sex, the weight of miniswine was similar to that of sex-matched control pigs (Fig. S2B,C).

### *CLN3^Δex7/8^* miniswine do not show signs of cognitive dysfunction but do exhibit robust motor abnormalities

To determine whether *CLN3^Δex7/8^* miniswine recapitulate the cognitive decline displayed in patients with CLN3 Batten disease (Jalanko and Braulke, 2009; Williams et al., 2006; Lamminranta et al., 2001; Jarvela et al., 1997; Kuper et al., 2018; Adams et al., 2006, 2007), animals were trained in a simple T-maze as previously described (White et al., 2018; Swier et al., 2022). Briefly, the miniswine were allowed to roam the maze for 4 days for 10 min each day during the acclimation phase. Then, the pigs were trained during the acquisition phase to select the food reward arms of the maze for ten 60 s trials on days one and two, during which the reward was placed repeatedly in the same arm of the maze. Finally, food was switched to the opposite arm (from the acquisition phase) during the reversal phase to test for the ability to relearn the new task. Wild-type and *CLN3^Δex7/8^* miniswine were tested on acquisition and reversal tasks when aged between 24 and 42 months of age but we did not detect sustained differences in performance (Fig. S3). Animals were not tested during the reversal phase at 42 months of age owing to poor performance in the acquisition tests at that age (accuracy on acquisition tests <80%; Fig. S3C,D), and tests using animals aged 48 months and older are not shown owing to the low number of animals (*n*=2/genotype).

To determine whether *CLN3^Δex7/8^* miniswine show motor deficits similar to those documented in children with CLN3 (Zimmerman et al., 2020; Ostergaard, 2021), gait of animals aged between 15 and 36 months was assessed on a pressure-sensor mat. For combined sexes, significant variables (*P*<0.05, Table S1) based on front foot position and pressure were used for synthesis of gait scores based on principal component analysis (PCA), similar to previously described protocols (Johnson et al., 2020; Swier et al., 2022). Male and female datasets were analyzed separately, using all 146 variables to synthesize gait scores on the basis of the Kaiser eigenvalue rule to reduce the dimensionality of these multivariate datasets. Based on scores for principal component analysis 1 (PCA1), *CLN3^Δex7/8^* animals had significantly altered gait compared to wild-type animals at every time point studied (Fig. 1B). The variables that contributed to the altered gait score in combined sex (Fig. 1C) and male datasets (Fig. S4A,B) were primarily associated with altered foot strike – i.e. footprint size and variability; the distance and variability associated with center-of-pressure (COP) trajectory for a single footfall. By comparison, female scores were composed largely of foot strike, spatial and balance variables, such as step length variability and swing percent ratio (Fig. S4F,G).

We next examined how the variables contributing to the gait scores are indicative of a phenotypic change over time. *CLN3^Δex7/8^* – primarily male – animals, consistently showed a smaller foot area (i.e. the area of the foot fall indicating that the entire foot is not in contact with the ground) than that of wild-type animals across the time studied (Fig. 1D; Fig. S4C). Importantly, small foot area was not a result of lesser body weight (Fig. S2), indicating differences in foot placement upon the mat in *CLN3^Δex7/8^* animals. COP distance and COP difference, i.e. variables that describe trajectory of a single footfall, were decreased in *CLN3^Δex7/8^* combined-sex datasets from animals aged 15, 18 and 36 months, indicating an altered balance phenotype (Fig. 1E,F). Similarly, male *CLN3^Δex7/8^* animals showed lower COP variance by 18, 24 and 36 months of age (Fig. S4D,E). Compared to female wild-type miniswine, female *CLN3^Δex7/8^* animals showed differences over the same time period, having a more-consistent step length (Fig. S4I), and a more-consistent placement of the right and left foot during the swing phase of the gait cycle (Fig. S4J). Taken together, these data indicate that the front feet of *CLN3^Δex7/8^* animals show altered stepping and COP dynamics, similar to the decreased stability observed in individuals with CLN3 disease (Ostergaard, 2021; Zimmerman et al., 2020).

The hind feet datasets were also assessed for significant variables for use in the PCAs. Overall, there were few consistent differences between genotypes over the time period studied, although – when combined – male and female *CLN3^Δex7/8^* animals did show altered gait patterns at 15 and 24 months of age, primarily related to greater efficiency of stance in males and less foot pressure in females (Fig. S5A-C). These differences are evident in principal component analysis 3 (PCA3) for combined sexes, and principal component analysis 2 (PCA2) for males and PCA1 for females. Activity was also assessed via a FitBark activity monitor in animals aged 24 to 48 months as previously described (Khanna et al., 2019). No difference in distance traveled, active/rest time or sleep quality was detected between genotypes (Fig. S6A-D).

### CLN3^Δex7/8^ miniswine exhibit visual decline and photoreceptor loss by 30 months of age

**Fig. 2.**
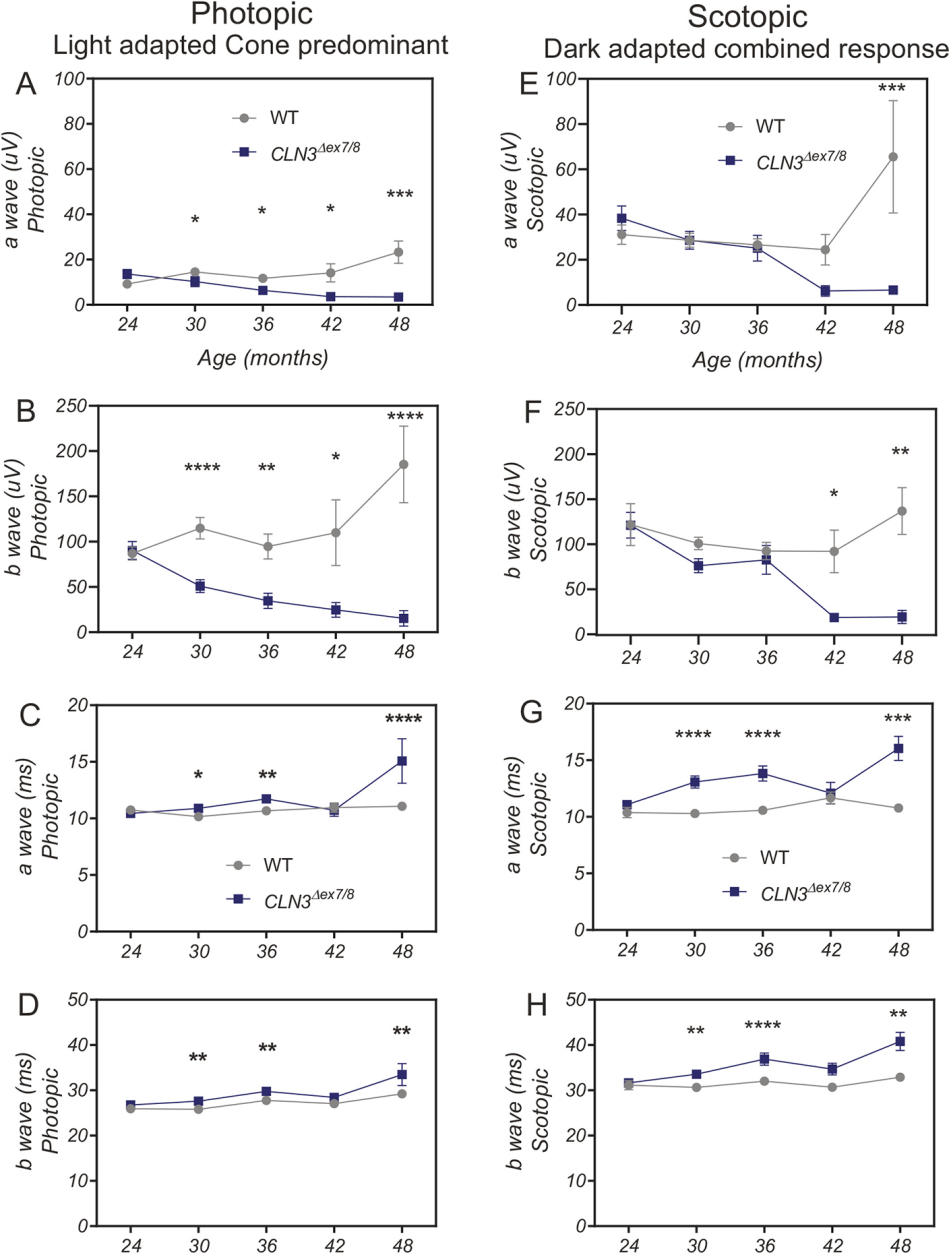
***CLN3^Δex7/8^* animals show photopic and scotopic visual deficits by 30 months of age as measured by electroretinography (ERG).** (A-D) Photopic data. At 30 months of age, *CLN3^Δex7/8^* miniswine begin to show a progressive decline in photopic a-wave (A) and b-wave (B) amplitudes. Latency delays arise at 30 months for both a-waves (C) and b-waves (D). (E-H) Scotopic data. Scotopic a-wave (E) and b-wave (F) amplitudes in *CLN3^Δex7/8^* miniswine begin to decline at 42 months of age and are extinguished at 48 months of age. Latency delays arise at 30 months for both a-wave (G) and b-wave (H) in all *CLN3^Δex7/8^* miniswine. At 42 and 48 months, WT *n*=two males; *CLN3^Δex7/8^ n*=3 (two males, one female). Mean±s.e.m. Two-way ANOVA, Fisher's LSD post-hoc test. *P≤0.05, **P≤0.01, ***P≤0.001, ****P≤0.0001. Photopic: 8.0 cd s/m^2^ flash at 2.0 Hz (cone predominant). Scotopic: 8.0 cd s/m^2^ flash at 0.1 Hz (bright flash standard combined response-mixed rods and cones). Absolute values for a-wave amplitudes are shown. Numbers of animals tested are listed in Table S4.

**Fig. 3.**
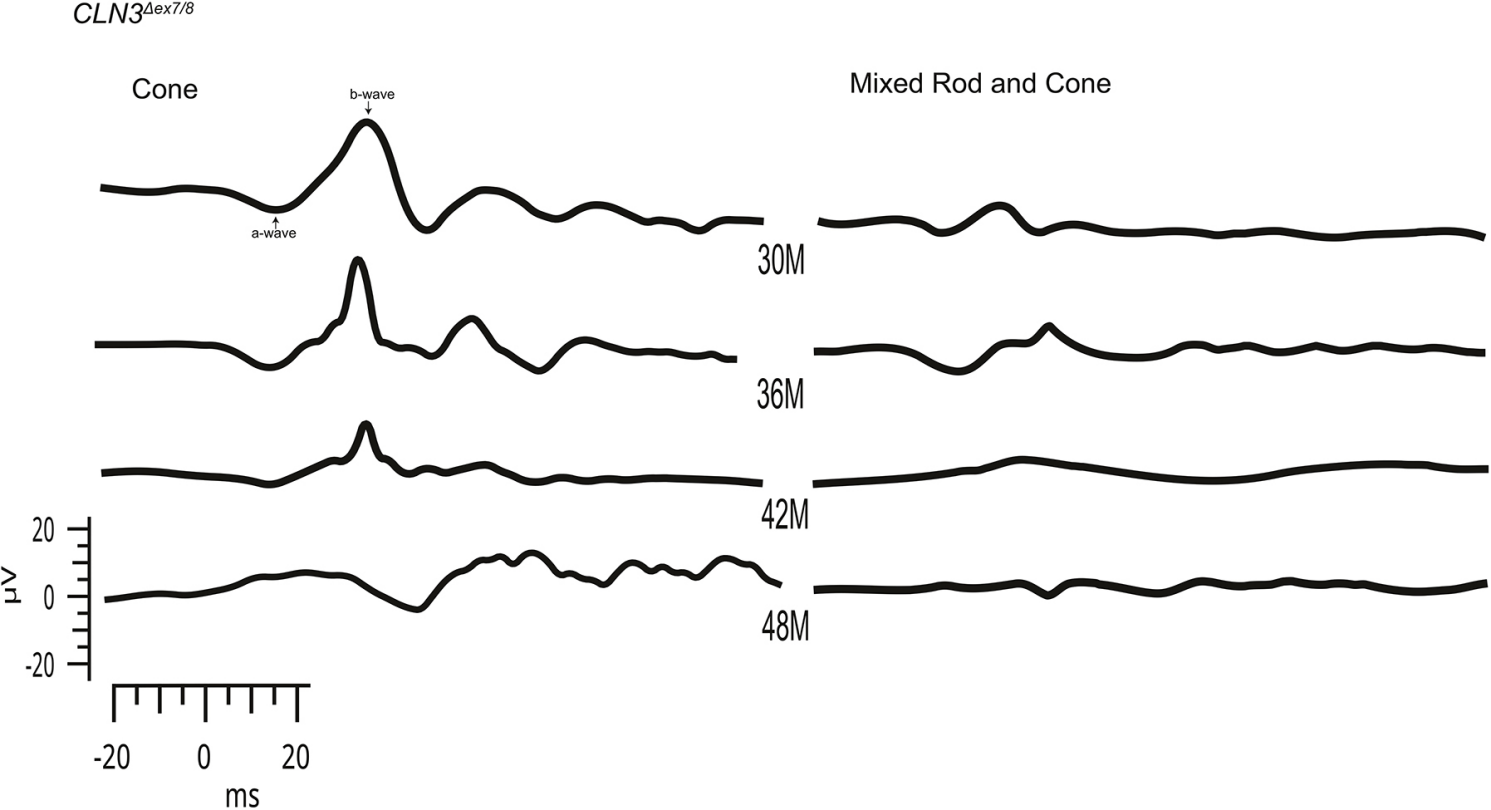
**In *CLN3^Δex7/8^* miniswine a- and b-waves reduce with age.** Mixed rod and cones responses are extinguished by 42 months (right); cone predominant responses are extinguished by 48 months. The decline in a- and b- amplitudes is readily seen in a- and b-waves of *CLN3^Δex7/8^* miniswine aged between 30 and 48 months. At 48 months, the cone predominant waveforms are completely extinguished, only background noise is recorded. At 42 months, the mixed rod and cone responses are nonrecordable. Photopic: 8.0 cd s/m^2^ flash at 2.0 Hz (cone predominant). Scotopic: 8.0 cd s/m^2^ flash at 0.1 Hz (bright flash standard combined response-mixed rods and cones). All wave recordings were obtained from the same *CLN3^Δex7/8^* male.

As vision loss primarily due to retinal degeneration is a characteristic phenotype of patients with CLN3 Batten disease (Volz et al., 2014; Staropoli et al., 2012; Kleine Holthaus et al., 2020; Zhong et al., 2020; Katz et al., 2008b; Collins et al., 2006; Kuper et al., 2021; Ku et al., 2017; Horiguchi and Miyake, 1992; Williams et al., 2006; Jarvela et al., 1997), we investigated whether *CLN3^Δex7/8^* miniswine show retinal degeneration. Animals were assessed using flash electroretinography (ERG) from 24 to 48 months of age. By 30 months of age, *CLN3^Δex7/8^* miniswine of both sexes showed reduced a-wave (photoreceptor response) and b-wave (bipolar cell response) amplitudes in a light-adapted, cone-responsive photopic protocol, with a- and b-wave responses declining over time (Fig. 2A,B). When we examined males and females separately, males showed reduced b-wave amplitudes at 30 months; however, reduced a-wave amplitudes were detected later at 42 months of age (Fig. S7A,B). No significant differences were detected in females (Fig. S7I,J). Delayed latencies regarding photopic a- and b-waves also arose in *CLN3^Δex7/8^* animals of both sexes at 30 months of age, consistent with widespread disfunction of remaining photoreceptors (Fig. 2C,D**)**. *CLN3^Δex7/8^* males also showed delayed latencies at 30 months of age in photopic b-waves but reduced latencies in a-waves at 36 months of age (Fig. S7C,D). Delayed a- and b-wave latencies were detected in *CLN3^Δex7/8^* females at 30 months of age, but this was not sustained (Fig. S7K,L). When measured in a dark adapted, mixed rod/cone-responsive scotopic ERG, *CLN3^Δex7/8^* miniswine of both sexes showed reduced a-wave amplitudes at 48 months of age and reduced b-wave amplitudes by 42 months age (Fig. 2E,F). *CLN3^Δex7/8^* males showed reduced scotopic a-wave amplitudes at 36 months of age and reduced b-wave amplitudes at 30 months of age (Fig. S7E,F). No significant differences were found in scotopic a- and b-wave amplitudes in females (Fig. S7M,N). Significantly delayed latencies in both waveforms arose by 30 months in both sexes of *CLN3^Δex7/8^* animals (Fig. 2G,H), suggestive of defects in photoreceptor neuronal transmission (Leinonen, 2016). In general, photoreceptor death results in loss of amplitude, while photoreceptor dysfunction results in delayed amplitudes. Delayed amplitudes often precede photoreceptor loss in retinal degenerations. Similarly, delayed scotopic a-wave latencies arose at 30 months in *CLN3^Δex7/8^* males and females (Fig. S7G,H; O,P); however, slight differences between the sexes were noticed in b-wave latencies, with delayed latencies at 36 months in males and 30 months in females (Fig. S7H; S7P). When examining the individual waveforms obtained from a single representative *CLN3^Δex7/8^* male between 30 and 48 months, we saw a reduction in the amplitude of both a- and b-waves that progressed until the waveform was completely extinguished by 48 months in the predominant cone responses, and completely extinguished by 42 months in the mixed rod and cone responses (Fig. 3).

**Fig. 4.**
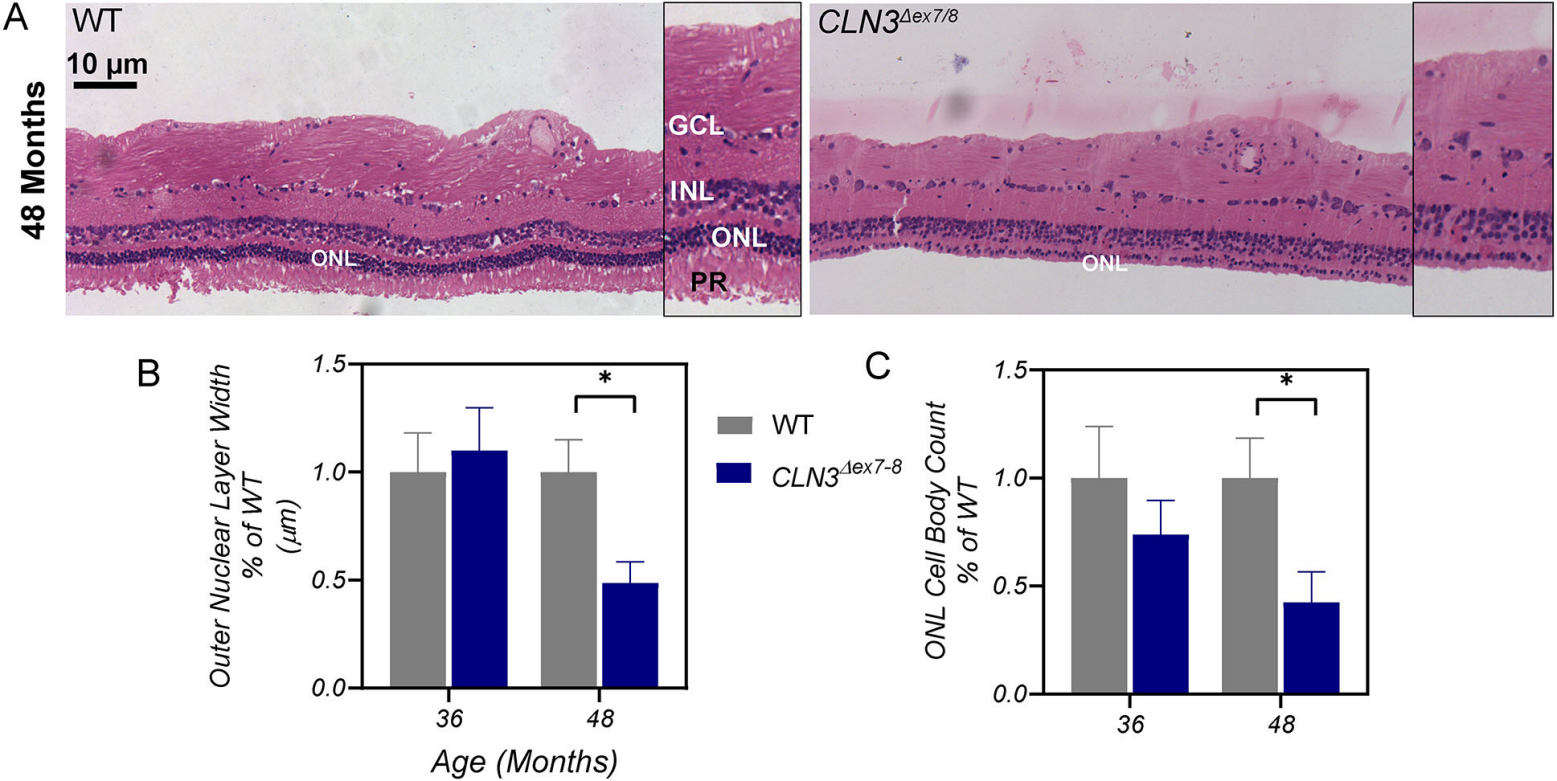
***CLN3^Δex7/8^* animals show retinal thinning as evidenced by loss of cells at 48 months of age.** (A) Comparison of retinal images from WT and *CLN3^Δex7/8^* miniswine aged 48 months. Loss of photoreceptors (PR) is seen in 48-month-old *CLN3^Δex7/8^* miniswine (right) compared with WT swine (left). Measured were retinas of 36 and 48-month-old miniswine. (B,C) Plotted is the percentage of the width of the outer nuclear layer (ONL) (A) and the ONL cell body count, i.e. loss of photoreceptors, (C) for WT and *CLN3^Δex7/8^* miniswine aged 36 and 48 months. At 36 months: *n*=5 (two females, three males); at 48 months: *n*=3 (one female, two males). Mean±s.e.m. Nested *t*-tests. *P≤0.05. GCL, ganglion cell layer; INL, inner nuclear layer.

Retinas were assessed for photoreceptor loss using Hematoxylin and Eosin staining and outer nuclear layer thickness and cell body measurements from 36 to 48 months of age. *CLN3^Δex7/8^* animals showed loss of photoreceptors at 48 months of age (Fig. 4A,C). Importantly, there is a complete loss of the photoreceptor layer in 48-month-old *CLN3^Δex7/8^* animals in the midperiphery of the retina (*n*=2 males; 1 female), indicating late-stage retinopathy by this time point, which correlates well with the greatly reduced (and almost extinguished) ERG amplitudes at 48 months.

### CLN3^Δex7/8^ miniswine display classic Batten disease pathology in several regions of the brain

**Fig. 5.**
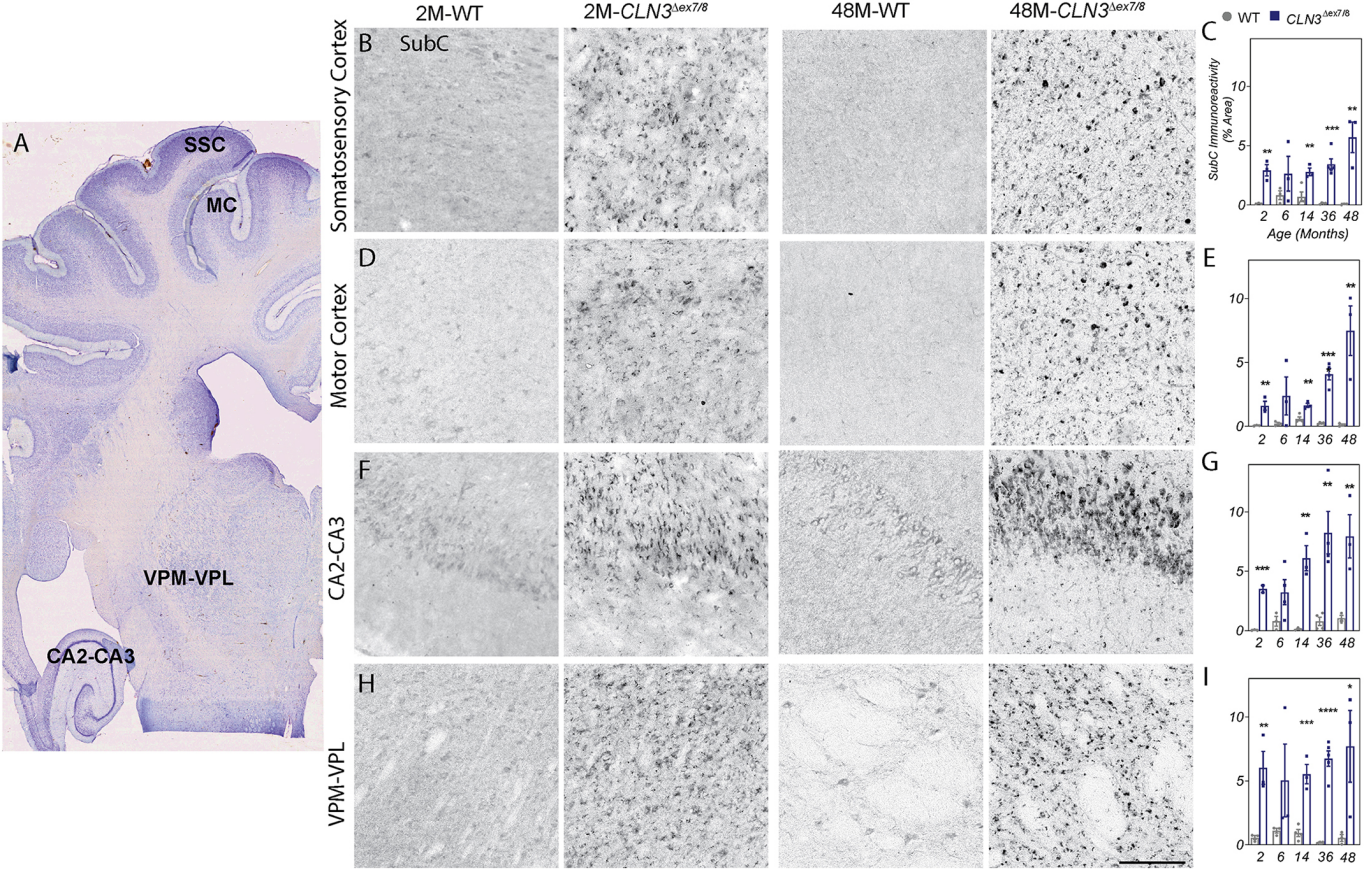
***CLN3^Δex7/8^* animals show accumulation of mitochondrial ATP synthase subunit C (SubC) in several brain regions.** (A) Nissl-stained coronal section of a miniswine brain indicating the anatomical location of somatosensory cortex (SSC), motor cortex (MC), ventral posteromedial and ventral posterolateral nuclei (VPM-VPL) of the thalamus, and CA2-CA3 of hippocampus. (B-I) Subunit C accumulation was evident in *CLN3^Δex7/8^* miniswine, beginning at 2 months of age in the somatosensory cortex (B,C), motor cortex (D,E), CA2-CA3 (F,G) and the VPM/VPL nuclei (H,I). Accumulation was persistent at all time points measured. Mean±s.e.m., unpaired *t*-test, *P≤0.05, **P≤0.01, ***P≤0.001, ****P≤0.0001. Scale bar: 200 µm. Numbers of animals tested are listed in Table S4.

To determine whether *CLN3^Δex7/8^* animals exhibit the pathological hallmarks of CLN3 disease, brains were excised, dissected and longitudinally examined for classic Batten disease histopathology in several brain regions. Specifically, the somatosensory cortex, motor cortex, ventral posteromedial (VPM) and ventral posterolateral (VPL) nuclei of the thalamus, and the hippocampal areas CA2-CA3 were assessed (Fig. 5A). Mitochondrial ATP SubC, a common component of Batten disease-associated lysosomal storage material, accumulated in *CLN3^Δex7/8^* miniswine by as early as 1 to 4 days of age (Fig. S8) and persisted at 2, 6, 14, 36 and 48 months of age in the somatosensory cortex (Fig. 5B,C), motor cortex (Fig. 5D,E), CA2-CA3 (Fig. 5F,G) and VPM/VPL nuclei (Fig. 5H,I).

**Fig. 6.**
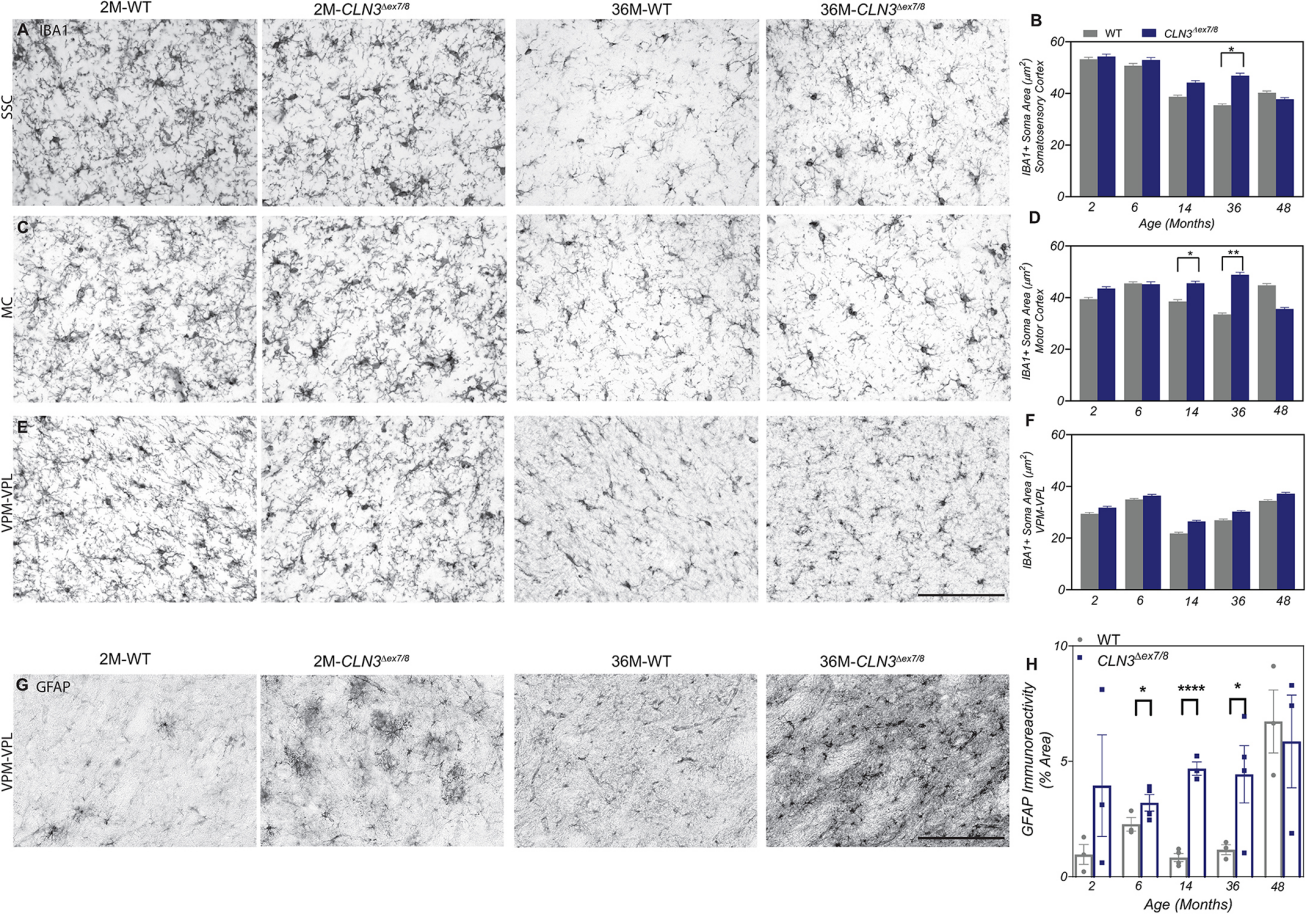
***CLN3^Δex7/8^* miniswine show microglia and astrocyte reactivity in several brain regions.** (A-F) Increased IBA1-positive (IBA1+) microglia soma size was evident at 14 to 36 months of age in the somatosensory cortex (A,B) and motor cortex (C,D) of *CLN3^Δex7/8^* animals. Microglial reactivity was not evident in ventral posteromedial and ventral posterolateral nuclei (VPM-VPL) (E,F). (G,H) GFAP-positive astrocytosis was evident between 6 and 36 months of age in the VPM/VPL of the thalamus of *CLN3^Δex7/8^* animals. Mean±s.e.m., nested *t*-test (IBA1) or unpaired *t*-test (GFAP), *P≤0.05, **P≤0.01, ****P≤0.0001. Scale bars: 200 µm. Numbers of animals tested are listed in Table S4.

In most forms of Batten disease, lysosomal storage and neuronal dysfunction are believed to initiate a neuroinflammatory cascade that results in the activation of glial cells. IBA1 (officially known as AIF1)-positive (IBA1+) microglia were examined for their activation status by measuring soma size as previously described (Davis et al., 2017). Within the cortex, the soma area was significantly enlarged in the somatosensory cortex of 36-month-old *CLN3^Δex7/8^* animals (Fig. 6A,B) and in the motor cortex of 14- and 36-month-old *CLN3^Δex7/8^* animals (Fig. 6C,D). Surprisingly, we detected no microglial reactivity in the VPM/VPL nuclei (Fig. 6E,F). Genotypes were then pooled together to determine whether there is a correlation between age and soma size or age and number of microglia. Smaller somas were identified in older animals (aged 14 and 36 months) in the somatosensory cortex, and numbers of microglia were found to be increased in younger animals (2 months) within the somatosensory cortex and the motor cortex (Fig. S9). Astrocyte reactivity was also examined, and although multiple cortical regions were investigated, strong phenotypic differences in glial fibrillary acidic protein (GFAP) immunoreactivity were only observed in the thalamus. Reactive astrocytes were first identified in the VPM/VPL nuclei in 2-month-old *CLN3^Δex7/8^* animals and were sustained longitudinally until 36 months (Fig. 6G,H).

In Batten disease, neurodegeneration is believed to occur as an end-stage result of neuronal dysfunction and neuroinflammation. To determine whether neurodegeneration does occur in the brains of *CLN3^Δex7/8^* animals, the thickness of the cortical plate was measured in sections labeled with the neuronal nuclei antigen (NeuN antigen; officially known as Rbfox3 in mammals). No overall cortical atrophy was detected in *CLN3^Δex7/8^* animals from 14 to 48 months (Fig. S10). Cortical thickness measurements may not be the most sensitive measure of overall cortical atrophy; hence, we proceeded with interneuron counts to detect possible neuronal loss. As particular populations of GABAergic interneurons have been shown to be selectively vulnerable in the cortex of CLN3 patients (Hachiya et al., 2006) and *Cln3^−/−^* mice (Mitchison et al., 1999, 2004), we also investigated the loss of cortical interneurons in the miniswine. Calbindin-positive (Calbindin+) neurons were examined in the deepest layers of the cortex (corresponding to layers 5 and/or 6). A significant reduction of calbindin+ interneurons was detected in *CLN3^Δex7/8^* animals at 6 months of age in the somatosensory cortex and 36 months of age in the motor cortex (Fig. S11), indicating that these cells may be lost progressively in *CLN3^Δex7/8^* animals.

## DISCUSSION

The *CLN3^Δex7/8^* miniswine model recapitulates many human phenotypes of CLN3 disease, including consistent retinal degeneration and gait abnormalities, as well as classic Batten disease pathologies, such as storage material accumulation, glial activation, photoreceptor loss, and calbindin interneuron loss in the somatosensory cortex and motor cortex. Importantly, when compared to *Cln3^Δex7/8^* mouse models, the *CLN3^Δex7/8^* miniswine model shows a more-severe and consistent phenotype of retinal cell loss and gait abnormalities.

In patients with CLN3 disease, vision loss is one of the first diagnosed symptoms, with the onset of visual failure occurring at a mean age of 6.4 years, with the mean age of diagnosis being 8.4 years of age (Collins et al., 2006; Kuper et al., 2021). In the *CLN3^Δex7/8^* miniswine, reduced ERG amplitudes and delayed latencies were first observed at 30 months of age, which – when scaled proportionally to the 15-year lifespan of the Yucatan miniswine model – equates to approximately the age of an older adolescent child. The range of onset does vary in CLN3 patients; males with an earlier onset have been diagnosed with the disease as late as 11.5 years (Collins et al., 2006). However, other studies have found a later (on average) onset of 29.7 years in males and females (Ku et al., 2017), which is reflective of the variability in the visual phenotype. In patients, ophthalmic examination may detect retinopathy, maculopathy, loss of retinal function as indicated by preferential loss of the dark-adapted ERG (scotopic) b-wave amplitude, reduction of scotopic a-waves amplitude and, eventually, reduction of light-adapted ERG (photopic) a- and b-wave amplitudes with a delay in b-wave responses as the disease progresses (Horiguchi and Miyake, 1992; Kuper et al., 2021; Weleber, 1998; Eksandh et al., 2000). We see similar trends in the *CLN3^Δex7/8^* miniswine, with a significant decrease in scotopic b-wave amplitude present before any reductions in scotopic a-waves, and an earlier reduction in photopic a- and b-waves that progressively declines at later stages of the disease. In contrast, *Cln3^Δex7/8^* mice models show b-wave declines at a much later stage of the disease that is akin to end-stage patients (Volz et al., 2014; Kleine Holthaus et al., 2020). Of note, at 48 months, we do see completely extinguished a- and b- waveforms in the light-adapted cone responses, and dark-adapted mixed rod and cone responses in both the left and right eye of *CLN3^Δex7/8^* males. Extinguished photopic and scotopic responses might not necessarily be indicative of complete blindness but, combined with the anatomic photoreceptor loss, strongly suggests that these male pigs are extremely visually impaired.

Similarly, optical coherence tomography (OCT) imaging in patients with an undetectable ERG response shows loss of photoreceptors, and marked atrophy of inner and outer nuclear layers of the retina (Wright et al., 2020; Preising et al., 2017). Our *CLN3^Δex7/8^* miniswine model also exhibited thinning and cell loss in the outer nuclear layer, and complete loss of photoreceptors in the oldest animals monitored (Fig. 4). In contrast, aged *Cln3^Δex7/8^* mouse models have shown only a loss of bipolar cells without retinal atrophy in the outer nuclear layer (Kleine Holthaus et al., 2020). Differences in photoreceptor localization between species might explain the phenotypic differences between *CLN3^Δex7/8^* miniswine and *Cln3^Δex7/8^* mice, as mice lack a macula region of concentrated photoreceptors, whereas swine have a macula-like region similar to that in humans (Shrader and Greentree, 2018).

We also investigated whether *CLN3^Δex7/8^* miniswine display gait abnormalities as a correlate with the loss of motor function, impaired balance or shuffling-gait phenotypes that are common in patients with CLN3 (Zimmerman et al., 2020). *CLN3^Δex7/8^* male miniswine presented a more-cautious gait with smaller footfalls and a controlled stance with lower COP distance values. COP measurements in gait analysis explain how much an individual moves the directional placement of a COP footfall without losing balance, and greater COP distance values indicate a lack of precise controlled movements (Lemay et al., 2014). Therefore, lower COP values might be indicative of a careful balancing strategy to compensate for lack of stability. Importantly, reduced variability in COP has been recorded in patients with neurological gait disorders and in patients recovering from stroke as an adaptation to avoid falls, and stabilize the gait (Bower et al., 2019; Ehrhardt et al., 2020). Whereas *Cln3^Δex7/8^* mouse models present with mild motor-coordination phenotypes that are difficult to recapitulate across laboratories, differences in *CLN3^Δex7/8^* miniswine gait were consistent across the time points studied. These differences were primarily male driven, echoing previous reports of sex-specific presentations and disease course in patients and mouse models (Kovacs and Pearce, 2015; Cialone et al., 2012). Interestingly, male *CLN3^Δex7/8^* miniswine had consistently smaller footfalls across the entire time period studied and – as there were no weight differences between genotypes in the male animals – smaller footfall is unlikely to be a result of body size differences. A possible explanation for small footfalls in *CLN3^Δex7/8^* miniswine could be the presence of osteoarthritis, which has been documented in patients with other lysosomal storage disorders, such as mucopolysaccharidoses (Borgo et al., 2018; Tomatsu et al., 2015), and presents in swine as a convex curvature of the knee that causes the principal digits of the foot to lift off of the ground (Jørgensen, 2000). This could result in smaller footfalls on a pressure-sensor mat, and more research is needed to understand the nuances of gait abnormalities in swine models of disease.

As observed in patients and *Cln3^Δex7/8^* mouse models, one of the most-common markers of Batten disease, i.e. mitochondrial ATP SubC, was found to accumulate throughout multiple brain regions in *CLN3^Δex7/8^* miniswine. However, unlike in patient and *Cln3^Δex7/8^* mouse models (Cotman et al., 2002; Pontikis et al., 2005), astrocytosis was only detected in the VPM/VPL nuclei of the thalamus and not in any examined cortical region of *CLN3^Δex7/8^* miniswine. One reason could be linked to the increased level of GFAP expression in white matter fibrous astrocytes compared to lower GFAP expression in gray matter protoplasmic astrocytes that has been documented in aging brains (Lundgaard et al., 2014). The latter might indicate the differential upregulation of inflammatory factors in activated white matter astrocytes that are not upregulated in gray matter astrocytes (Guillot et al., 2015). In other miniswine models of neurodegeneration GFAP-positive astrocytes have also not been detected within cortical gray matter (Ardan et al., 2019), possibly indicating an even greater differential regulation of GFAP expression in the protoplasmic astrocytes of swine (Ardan et al., 2019). Additionally, gene expression studies in astrocytes have found species-specific differences between humans and mice, increasing the likelihood that miniswine also have species-specific astrocyte expression profiles that are, perhaps, not represented through astrocyte labeling as commonly used in research of Batten disease (Li et al., 2021).

Although activated astrocytes were not present in the cortex, we did find activated microglia with enlarged somas in the somatosensory and motor cortices in *CLN3^Δex7/8^* miniswine. Activated microglia have been identified in *Cln3* mouse models in multiple cortical regions (Pontikis et al., 2004) and microglia isolated from *Cln3^Δex7/8^* mice have been shown to be primed for activation (Xiong and Kielian, 2013). Interestingly, two different populations of microglia have been isolated from *Cln3^Δex7/8^* mice – one showing autofluorescence and increased levels of LAMP1, and one not showing autofluorescence or increased levels of LAMP1– indicating lysosomal disfunction in microglia with autofluorescence (Burns et al., 2020). Autofluorescent microglia also show increased rates of cell death (Burns et al., 2020), indicating that accumulated autofluorescence functionally impacts microglia by reducing the ability to remove debris, thus contributing to the pathology of *CLN3* disease. Accumulation of mitochondrial ATP SubC (indicative of autofluorescence accumulation) in the cortices of the *CLN3^Δex7/8^* miniswine might explain the enlarged somas, as the microglia are changing to the amoeboid phagocytizing state to remove excess autofluorescent material.

Although swine models are touted to be more translatable to conditions of Batten disease in humans, species differences still exist. For example, swine do not present with ulceration of the gastrointestinal tract in stress induced environments and, although the connective tissue of liver in swine is anatomically similar to that in humans, swine do not have the same susceptibility to injury-induced cirrhosis of the liver (Swindle et al., 2012). Additionally, the use of swine and other large animal models of disease are complicated by the lack of species-specific tools, such as antibodies, protocols, behavior equipment and tissue atlases. Regarding central nervous system-specific disease modeling, brain collection is challenging owing to the thicker and denser skull in swine, MRI atlases are not readily available for imaging studies and neurocognitive tests may be difficult to perform or interpret. While there are several caveats and while improvements need to be made in the use of large animal models of disease, the *CLN3^Δex7/8^* miniswine model is a marked improvement over the traditional *Cln3^Δex7/8^* mouse model. Miniswine are similar in size, anatomy, and physiology to the human condition, and the *CLN3^Δex7/8^* miniswine model presents with robust visual and gait impairments that are typically absent from CLN3 mouse models. *CLN3^Δex7/8^* miniswine, therefore, hold the promise of improving our understanding of CLN3 disease mechanisms and provide a more-relevant setting in which to test therapeutic interventions.

### Future considerations

We employed a simple T-maze to discover whether the *CLN3^Δex7/8^* miniswine recapitulate the learning and/or memory deficits seen in patients. At 24 months, when the animals were first tested, delays in learning a new task were seen in only male *CLN3^Δex7/8^* miniswine; however, in subsequent trials, these delays disappeared. As the animals aged, they either became conditioned to the maze layout or their larger sizes reduced maneuverability within the maze, and after the second or third trial any novel differences disappeared. A simple T-maze may still be an effective tool for neurocognition testing in swine, as other neurology studies have found significant reversal deficits when using younger, smaller animals (Singh et al., 2019); and we have shown significant learning delays in our previously published miniswine models in animals aged 9-12 months (White et al., 2018; Swier et al., 2022).

Other large-animal Batten models have shown progressive declines in T-maze performance, and testing was discontinued when animals were unable to navigate the maze, due to either vision loss or motor/behavioral declines (Mitchell et al., 2018; Katz et al., 2014). The *CLN3^Δex7/8^* miniswine were able to navigate through the maze and make selections, even after showing signs of severely diminished ERG amplitudes, possibly due to the recall of spatial memory acquired during previous T-maze testing (Mitchell et al., 2018). The time it takes to navigate and the velocity of the body moving through the maze should be included in future testing to determine whether transit time is correlated with diminished vision.

For phenotype monitoring in the miniswine, we wanted to use a tracking device that would record daily activities not influenced by frequent human interactions. For this, we used the FitBark 2 attached to a collar around the miniswine neck. This device was well tolerated around the neck, and has provided very useful data in previous miniswine studies (Khanna et al., 2019) as well as other translational animal models, such as the ovine osteoarthritis induction and canine Alzheimer dementia-like syndrome models (Newell et al., 2018; Valenzuela et al., 2022). In these other animal studies, the FitBark 2 device was worn by the animals for at least two weeks (a maximum of 3 months in the canine model), compared to one week in our study. Future longitudinal miniswine studies are needed to track a longer time course that might improve data quality and reduce variability.

Although there are limitations to the *CLN3^Δex7/8^* miniswine model, we do see a robust gait phenotype, a retinal phenotype documented by cellular loss and ERG dysfunction, and the accumulation of autofluorescent storage material in various regions of the brain. The glial phenotype is not as robust but does correlate with previously published studies supporting the genesis of neuroinflammation in the thalamus (Kielar et al., 2007), supporting the earlier proinflammatory state of microglia before neuronal deficits (Xiong and Kielian, 2013), and a tendency for hypertrophic, active microglia to be associated with progressive Batten disease (Vuillemenot et al., 2015; Parviainen et al., 2017). Keeping these limitations in mind, therapeutic testing in the CLN3 miniswine would need to focus on storage material accumulation and astrocyte activation in the thalamus for early pathological markers of efficacy (∼6 months), and microglia activation in the cortex and gait assessment of the front legs for later efficacy results (∼15 months). Of note, the later retinal phenotype (∼30 months) in the *CLN3^Δex7/8^* miniswine may pose a challenge for testing therapeutics in the eye if studies take a more traditional approach. When using large animal models for preclinical research, we need to consider using a different toolbox to clearly map out the phenotype. Recent studies have demonstrated the use of neurological clinical scoring (Eaton et al., 2022) and clinical tools such as OCT (Cheng et al., 2018; Xie et al., 2018) as more translational tools for efficacy testing. By using an animal model that is physiologically and anatomically similar to humans should increase the likelihood that a treatment will be useful and, eventually, be approved for clinical trials. Ultimately, we are all trying to find better therapeutics that will successfully treat individuals with Batten disease.

## MATERIALS AND METHODS

### Study approval

All animals were maintained at Precigen Exemplar (Sioux Center, IA, USA) under an approved institutional animal care and use committee (IACUC) protocol (MRP2015-005).

#### Targeting vector construction

Genomic DNA was isolated from Yucatan miniswine fetal fibroblasts. A 11.2 kb PCR product that included the *CLN3* gene region from exon 3 to exon 9 was amplified using a high-fidelity polymerase (Platinum Taq High Fidelity, Invitrogen) and the *CLN3* primers pCLN3F3 and pCLN3R3 (Table S2) under the following conditions: one cycle of 94°C for 3 min, 35 cycles of 94°C for 20 s, 56°C for 10 s, 68°C for 15 min and, finally, one cycle of 68°C for 3 min. The PCR product was subcloned into pCR2.1-TOPO (Invitrogen) and sequenced. All DNA sequencing was performed by the University of Iowa DNA Facility. The resulting plasmid served as the template for PCR amplification of the 5′-3′ homologous targeting arms that were subcloned sequentially into a plasmid containing a PGK-NeoR cassette. The primers for the 5′ arm were CLN3 5′armR(EcoRV)2 and CLN3 5′armF(XhoI)2. The primers for the 3′ arm were CLN3 3′armF(HindIII)2 and CLN3 3′armR(HindIII)2 (Table S2). PCR for the 5′ arm was performed using PfuUltra (Agilent Technologies) under the following conditions: one cycle of 95°C for 3 min, 35 cycles of 95°C for 20 s, 65°C for 20 s, 72°C for 4 min and, finally, 72°C for 3 min. PCR for the 3′ arm was performed using Platinum Taq High Fidelity (Invitrogen), under the following conditions: one cycle of 94°C for 3 min, 35 cycles of 94°C for 20 s, 62°C for 20 s, 68°C for 3 min 40 s and, finally, one cycle of 68°C for 3 min (Table S2).

### rAAV production

PCR amplification of a 4.5-kb amplicon from the plasmid described above was achieved by using the following primers: AAVCLN3NeoRF(NotI) and AAVCLN3NeoRR2 (Table S2). This product was subcloned into the recombinant adeno-associated virus (rAAV) proviral plasmid pFBAAV2-CMVP.NpA (obtained from University of Iowa Gene Transfer Vector Core) and transfected into and grown in SURE 2 cells (Stratagene). The rAAV was produced by the University of Iowa Viral Vector Core Facility.

### Fetal fibroblast infection and selection

Passage zero male Yucatan fetal fibroblasts (1×10^6^) were infected with rAAV carrying the *CLN3*-targeting construct as described by Rogers et al. (2008a). After 24 h, cells were detached with trypsin and plated on 96-well collagen-coated plates. Selection was initiated 48 h later with the aminoglycoside antibiotic G418 (100 μg/ml; Corning, 30-234-CI). Ten to 13 days later, each infected cell plate was split into three 96-well plates (one plate for freezing, one for propagation and one for immediate PCR screening).

#### PCR screening and cell handling

Following selection, ∼40% of wells contained live cell colonies. Cells were subjected to 5 μl lysis buffer comprising 50 mM KCl (Sigma), 1.5 mM MgCl2 (Sigma), 10 mM Tris-HCl (RPI Corp) pH 8.5, 0.5% Nonidet P-40 (Amresco), 0.5% Tween (RPI Corp) and 400 μg/ml proteinase K (Qiagen), and were then incubated at 65°C for 30 min, followed by 95°C for 10 min. Primers Screen R (NeoR), pCLN32PCRF1 and pCLN3PCRR18 (Table S2) were used to PCR amplify 2 μl of lysate under the following conditions: one cycle of 2 min at 94°C, 30 cycles of 94°C for 20 s, 58°C for 20 s, 68°C for 3 min 10 s and, finally, one cycle of 68°C for 3 min. The wild-type allele yielded a product of 2.7 kb, while the expected product for the targeted allele was 1.8 kb. The PCR-positive cells were grown to 100% confluence and either infected with rAAV-Cre or cultured further for DNA isolation.

### Excision of the NeoR cassette

PCR-positive fetal fibroblast cells were infected with rAAV-CMV-Cre. Three to 6 days later, 90% of infected cells were frozen and the remaining cells propagated for PCR characterization. Cells were lysed in 5 μl lysis buffer as described above, and excision of the selectable marker was detected by PCR using primers pCLN32PCRF1 and pCLN33PCRR18 (Table S2) under the following conditions: one cycle of 2 min at 94°C, 35 cycles of 94°C for 20 s, 58°C for 20 s, 68°C for 4 min and, finally, one cycle of 68°C for 7 min.

### Southern blotting

To validate PCR-positive cell lines, genomic DNA was isolated (Gentra, Qiagen) from fibroblasts grown on the propagation culture dishes. Two to 10 ng of genomic DNA was whole-genome amplified (Repli-G, Qiagen) and digested with AflII and SspI. Following gel electrophoresis, samples were transferred to a positively charged nylon membrane (Roche Diagnostics) by using an alkaline transfer procedure. The membrane was briefly rinsed in 5× saline-sodium citrate (SSC) buffer, completely dried and subjected to UV crosslinking. The DNA probes for *CLN3* and the NeoR cassette were produced by PCR amplification using primers pCLN3probeF2/pCLN3probeR3 and NeoR-F/NeoR-R, respectively (Table S2). Probes were radiolabeled with [α-^32^P]dNTP by random priming using the Prime-a-Gene Labeling System (Promega), and the radioactive probes were purified using CHROMA SPIN+TE-100 columns (Clontech). Membranes were prehybridized in Rapid-hyb Buffer (Cytiva Amersham) for 30 min at 65°C; then, 25 μl of α-^32^P-labeled probe was added and hybridization proceeded at 65°C for 2 h. The membrane was washed once in 2×SSC and 0.1% SDS at room temperature for 20 min and three times in 0.1× SSC and 0.1% SDS at 65°C for 15 min each. To confirm the animal genotype, high-molecular weight genomic DNA was isolated from miniswine umbilici. The remaining steps were performed as described above.

### Somatic cell nuclear transfer

Nuclear transfer was performed by Trans Ova Genetics (Sioux Center, IA, USA) as previously described (Walker et al., 2002). Embryo transfer was performed at Precigen Exemplar. Briefly, reconstructed oocytes were transferred into synchronized post-puberty domestic gilts on the first day of standing estrus. Recipient gilts were pre-anesthetized intravenously with propofol (0.5-5 mg/kg), and anesthesia was maintained with inhaled isoflurane (3-5% in oxygen via face mask). Following a midline incision to access the uterus, reconstructed embryos were transferred into the oviduct at the ampullary-isthmus junction. Intra- and postoperative analgesia was provided by intramuscular injection of flunixin meglumine (2.2 mg/kg). Recipient animals were checked for pregnancy by abdominal ultrasound after day 21 and throughout gestation.

### Phenotype monitoring

Wild-type and *CLN3^Δex7/8^* miniswine were monitored from 24 to 48 months of age. Twenty-nine animals were monitored up to 36 months of age (wild type: nine male, six female; *CLN3^Δex7/8^*: eight male, six female) and five animals were monitored up to 48 months of age (wild type: two male; *CLN3^Δex7/8^*: two male, one female). Throughout the study, animal husbandry staff monitored animal health by visually inspecting the accumulation of fat around the animal, using body score levels between 1 and 5, with 1=emaciated/unhealthy and 5=overweight (Muirhead and Alexander, 1997). Also monitored were weight and the development of Batten disease phenotypes, such as signs of vision loss (i.e. running into pens, walls, gates; loss of visual tracking), seizures/muscle spasms, walking impairments (i.e. off-balance, poor posture, hypermetria), and lethargy, abnormal feeding behavior and assessment of social behavior (i.e. type and frequency of vocalizations, interactions with pen mates). Age of disease onset was recorded when abnormal phenotypes were detected.

### FitBark activity monitoring

Miniswine were tracked every 6 months from 24 to 48 months of age over the course of 6 days by using a FitBark 2 device attached on the neck using a common dog or calf collar as previously described (Khanna et al., 2019; Swier et al., 2022). Distance traveled, active time, rest time and sleep quality were analyzed with GraphPad Prism 8.0 using a two-way ANOVA, uncorrected Fisher's least significant difference (LSD) post-hoc test.

### Simple T-maze

Memory and learning capabilities were monitored using a simple T-maze as previously described (White et al., 2018; Swier et al., 2022), with the exception of using dry animal pellets as a reward. Briefly, during the acquisition phase (first 2 days of testing), animals learned in which arm a food reward was located. During the reversal phase (second 2 days of testing), the reward was moved to the opposite arm and animals had to relearn the location of the reward. Tests were video recorded and tracked with AnyMaze software v4.99 (Stoelting Co. Wood Dale, IL, USA), and observer unaware of the experimental conditions watched the T-maze videos and recorded the arm chosen by each animal during each phase. The number of correct responses were analyzed with GraphPad Prism 8.0 using a mixed-model ANOVA with Sidak's multiple comparisons.

### Gait assessment and principal component analysis

Motor performance was analyzed using a pressure-sensor mat as previously described (Swier et al., 2022; Beraldi et al., 2015). Briefly, animals were trained to walk on a 4.87×0.6 m Zeno Electronic Walkway (ZenoMetrics Peekskill, NY, USA) every 3 months from 15 months to 24 months of age, and every 6 months from 24 to 36 months of age. Five walks per subject were analyzed at each time point, with each walk consisting of eight consecutive footprints (a total of 40 steps per animal).

Front and hind feet data were processed with PKMAS Software ver. 509C1 (Protokinetics LLC, Havertown, PA, USA), and 146 variables were collected per footprint. The 146 variables include the mean, standard deviation (SD) and coefficient of variation (% CV) for 13 main parameters, categorized by: foot strike (i.e. integrated pressure, foot area, stance COP distance, and stance COP path efficiency), spatial orientation (i.e. step length absolute step length, stride width and stride length), temporal orientation (i.e. step tep time, stride time and stride velocity) and balance (i.e. stance percentage of gait cycle and swing percentage of gait cycle). The PKMAS software only obtains the means for total velocity, walk ratio and cadence. (Table S3). At first, all 146 variables were used to generate principal components for the combined-sex dataset. With this method, we found at least two dimensions that separated the animals by genotype. One dimension separated females only, whereas a different dimension separated males only. After removing parameters that contribute <1% to the principal component [based on the Kaiser rule, parameters are selected if their corresponding eigenvalues are >1 (Kaiser, 1960)], we reduced the number of variables for the second principal component from 146 to 33 parameters. However, we still found the separation by sex in different dimensions. Therefore, at each time point, *t*-tests were run on each of the 146 variables within combined sexes to determine which variables were significantly different at *P*≤0.05. When a variable was significant in at least two time points (indicating relevance to a phenotype), this variable was included in a principal component analysis (PCA) implemented in R utilizing the FactorMineR package (Lê et al., 2008) to determine which PCAs explained the most variation in the data. Using this method for parameter selection, resulted in a principal component with at least one dimension, explaining the variation between genotypes for both males and females (not separated by sex). The same method was used for the selection of hind-gait variables, however *t*-tests did not reach significance within at least two different time points, hence the alpha value was increased to *P*≤0.1 for the hind-foot datasets.

As we saw a separation in principal component dimension by sexes when running all 146 parameters, we ran two more principal components (one with females only, and another with males only). For female principal components, the first principal component (using the Kaiser rule) reduced the parameters from 146 to 23. The second principal component was run with only these 23 parameters – all of which had corresponding eigenvalues of >1) – resulting in factors that explained the variation between the two genotypes over multiple longitudinal time points (Table S1). For male principal components, the first principal component (using the Kaiser rule) reduced the parameters from 146 to 39. The second principal component was run with only these 39 parameters, resulting in a principal component with only one statistically significant dimension – which explained the variation between genotype – but only 21 parameters in the sample correlation matrix had eigenvalues of >1. Hence, the third principal component was run with these 21 parameters and we started to see dimensions that separated the genotypes across longitudinal time points. However, the sample correlation matrix for the third principal component continued to have multiple parameters that contributed <1% to the principal component, so we continued onto a fourth and final principal component with only 17 parameters (Table S1). This final principal component separated the two genotypes (*P*= 0.000002) and also separated over multiple longitudinal time points.

#### Gait score

*t*-tests were calculated for each principal component between wild-type and *CLN3^Δex7/8^* miniswine using pooled time points to determine which principal component significantly separated by phenotype at *P*≤0.05, and the significant principal component was used to graph the overall gait score. Principal component gait scores were standardized to the earliest wild-type average (15 months of age) and, thus, the average control score is equal to zero (no deficits). Data were then analyzed in GraphPad Prism 8.0 using a mixed-model ANOVA with uncorrected Fisher's LSD post-hoc test.

### Electroretinogram

Miniswine were tested for retinal function using a flash electroretinogram (ERG) every 6 months from 24 to 48 months of age as previously described (Swier et al., 2022). Our dark-adapted ERG experiments were performed after 20 min of dark adaptation, with external light eliminated, shades over all light sources and a piece of blackout fabric over the eyes of the pig while being anesthetized. We used only a dim red light as needed to set up the test. Because we carefully controlled light exposure to the eyes of the pig, we did not measure ambient light in the room. Light-adapted testing was performed after at least 10 min exposure to artificial light, i.e. standard operating room illumination. For the procedure, animals were anesthetized with 14 mg/kg ketamine (intramuscular) and anesthesia was maintained with 1-2% isoflurane. One drop of 1% Tropicamide ophthalmic solution (according to the quality standards of the United States Pharmacopeia (U.S.P.)] was placed in each eye to cause dilation. Reference electrodes were connected to each ear and a ground electrode to the midline forehead. One drop of 0.5% proparacaine hydrochloride ophthalmic solution U.S.P. was placed in each eye as a local anesthetic and eye speculums were placed inside each eye to fix the eyelids open. The smaller sticky pads of a DTL Plus Electrode (LKC Technologies) were attached to the rostral side and larger sticky pads to caudal side of each eye. Each respective DTL electrode (right and left) was connected to the respective extension lead of the right or left reference electrode, and the ground and both reference electrodes were connected to a RETeval device (LKC Technologies).

The rabbit/minipig, photopic 2-step light-adapted protocol was used for each eye and produced an 8.0 cd s/m^2^ flash at 2.0 Hz flash followed by an 8.0 cd s/m^2^ flicker at 28.3 Hz. After photopic testing of both eyes, all lights in the room were extinguished, the RETeval device was calibrated for dark adaptation and animal were allowed to adapt to the dark for 20 min. After dark adaptation, the rabbit/minipig, scotopic 4-step protocol was used for each eye. The first step produced a 0.06 cd s/m^2^ flash at 0.5 Hz (dark adapted rod only response), followed by an 8.0 cd s/m^2^ flash at 0.1 Hz (dark-adapted mixed-rod and cone response), followed by a 25 cd s/m^2^ flash at 0.05 Hz (dark-adapted mixed-rod and cone response to higher intensity flash). Raw (unsmoothed) data values were used to calculate amplitudes. The a-wave amplitude was recorded as the pre-stimulus baseline to an a-wave trough, and the b-wave amplitude was measured from an a-wave trough to the highest waveform peak. Amplitude data from left and right eyes, as well as latency data from left and right eyes were pooled together for each genotype/time point. The a- and b-wave amplitudes/peak times for 8.0 cd s/m^2^ photopic flash responses and 8.0 cd s/m^2^ scotopic mixed rod/cone responses were analyzed using GraphPad Prism 8.0 and a two-way ANOVA, uncorrected Fisher's LSD post-hoc test.

### Tissue collection and processing

For histopathological assessment animals were sacrificed with pentobarbital at 2, 6, 14, 36 and 48 months of age. One hemisphere of the brain was placed into 10% neutral buffered formalin (∼3 weeks) and subsequently sub-dissected into cortex, hippocampus, and thalamus blocks. Blocks were equilibrated in cryoprotectant solution (30% sucrose in TBSA) at 4°C. Blocks were serial sectioned (50 µm) on a freezing microtome (Leica) and free-floating sections from the somatosensory cortex, motor cortex, VPM/VPL nuclei of the thalamus and the hippocampus were placed in 6-well plates for immunohistochemistry.

Eyes were placed in 10% neutral buffered formalin (∼3 weeks) and retinas were removed. Retinal structure was separated from the eyecup and dissected to locate the midperiphery region of the retina. These dissected tissues were further fixed in 4% paraformaldehyde in PBS for 1 h at room temperature in glass containers. After secondary fixation, they were washed in PBS and further processed for plastic embedding during steps of dehydration, infiltration and, finally, embedded using the Technovit 7100 kit (Electron Microscopy Sciences, 14653). Briefly, retinal tissues were taken through increasing dilutions of ethanol (i.e. 50% for 2 h at room temperature, 70% overnight at 4C°, 80% for 1 h on ice, 95% for 1 h on ice and 99% (absolute) for 1 h on ice), then 99.9% (pure) acetone 1 h on ice for dehydration, and washed a final time in 99% ethanol 1 h. Then, for Technovit infiltration retinal tissues were incubated in 1:1 dilution of Technovit Infiltrate/ethanol (99%) overnight at 4C°. The next day, retinal tissue was placed in Technovit Infiltrate for two changes (each 30 min) on ice and kept in Technovit Infiltrate overnight at 4C°. On the day of embedding, retinal tissues were placed again in Technovit Infiltrate on ice for 1-2 h. Finally, retinal tissues were placed in polymerization solution for 15 min on ice while preparing the plastic molds to embed the tissue. When ready to embed, polymerization solution was half pipetted into the embedding cavity of the mold and, using forceps, retinal tissues were carefully placed in the desired orientation for cutting. The cavity was then filled up with polymerization solution and a mounting block was positioned. Embedded tissues were stored at room temperature overnight to allow complete polymerization. Thereafter, 3 µm sections were made using a microtome. After sections were dried overnight, they were stained using Multiple Stain Solution (Polysciences, 08824-100) for 2 min, washed with 70% ethanol, and mounted with permount (Fisher Scientific, SP15-500).

### Immunohistochemistry, microscopy, and analysis

Immunostaining for free-floating sections followed previous methods (Bible et al., 2004). The following primary antibodies and their dilutions were used: anti-ATP synthase subunit C (Abcam, ab181243; 1:2000), anti-GFAP (Dako, Z0334; 1:16,000), anti-IBA1 (BioCare Medical, 290; 1:2000), anti-NeuN (Millipore Sigma, MAB377; 1:2000), and anti-Calbindin (Swant, CB38; 1:2000). Secondary antibodies were biotinylated goat anti-rabbit or biotinylated goat anti-mouse depending on the host of the primary antibody. Secondaries were detected with an avidin/biotin blocking kit (Vector Laboratories, ABC-HRP, PK-4000), followed by incubation in 3,3′-diaminobenzidine (DAB). Sections labeled for mitochondrial ATP SubC, GFAP and NeuN were scanned using an Aperio Versa slide scanner (Leica Biosystems, IL, USA) at 20× magnification. At least three images were extracted from the somatosensory cortex, motor cortex, hippocampus and VPM/VPL nuclei of the thalamus using Aperio ImageScope 12 software (Leica Biosystems), and images were condensed by Adobe Photoshop and split into RGB channels by ImageJ (NIH) (Schneider et al., 2012). For mitochondrial ATP SubC and GFAP, total percent area was measured with adjusted threshold and analyze particles settings in ImageJ. For NeuN, cortical plate thickness was measured in coronal sections of the somatosensory and motor cortex following methods previously published (Poppens et al., 2019). IBA1- and calbindin-labeled sections were imaged on a Nikon 90i microscope (Nikon instruments, Inc.) at 20× magnification, images condensed with Adobe Photoshop, and split into RGB channels with ImageJ. For IBA1 and calbindin, soma size and number were measured by adjusting the threshold and particle size settings in ImageJ software (NIH). For each path point, we examined three sections per brain region per animal. Three images or measurements were taken for each section, resulting in nine technical replicates per brain region per animal.

Retinal sections from each animal were imaged at 10×, 20× and 40×. Inner and outer nuclear layers were measured in 10× retinal images with ImageJ, measuring the thickness of each layer in triplicates using the line tool in NIS-Elements. From wild-type animals, we obtained 60 images at 36 months and 24 images at 48 months; the number of images obtained from *CLN3^Δex7/8^* animals was 68 at 36 months and 36 at 48 months. Measuring the inner nuclear layer proved to be more difficult in 48-month-old animals, due to histological artifacts in some of retinas, yielding a data set too small for analysis (*n*=1/genotype). Additionally, thickness of the outer nuclear layer was quantified by counting the number of photoreceptor cells (number of nuclei) stacked in the outer nuclear layer in triplicates from each 20× image (≥5 for each animal). ImageJ software was used to mark nuclei that were counted and, prior to quantification, images were randomized for both age and sex of the animal to diminish bias. For each image, counts were taken from the middle, left and right areas of the retina presented in each image, making efforts to stay consistent with each region from image to image.

### Statistics

All data analyses were performed with GraphPad Prism 8.0 or equivalent. Outliers were removed using the ROUT method with Q=1. For each time point, unpaired *t*-tests were used to compare the means (percent total area) for mitochondrial ATP SubC and GFAP, the means for cortical plate thickness, and the mean number of calbindin+ cells between *CLN3^Δex7/8^* and wild-type miniswine. For each time point, nested *t*-tests were used to: compare IBA1+ soma sizes per animal; and retinal layer thickness per animal. Detailed statistical tests are described in the figure legends. *P<0.05, **P<0.01, ***P<0.001, ****P<0.0001. Animal numbers for behavior testing are listed in Table S4.

## Supplementary Material

10.1242/dmm.050038_sup1 Supplementary information
