# Supplementary material for: A novel porcine model of CLN3 Batten disease recapitulates clinical phenotypes
Source: Dis Model Mech. 2023 Aug 7;16(8):dmm050038. doi: 10.1242/dmm.050038 (PMC10434985; doi:10.1242/dmm.050038)
Supplement: Supplementary information [file dmm-16-050038-s1.pdf]

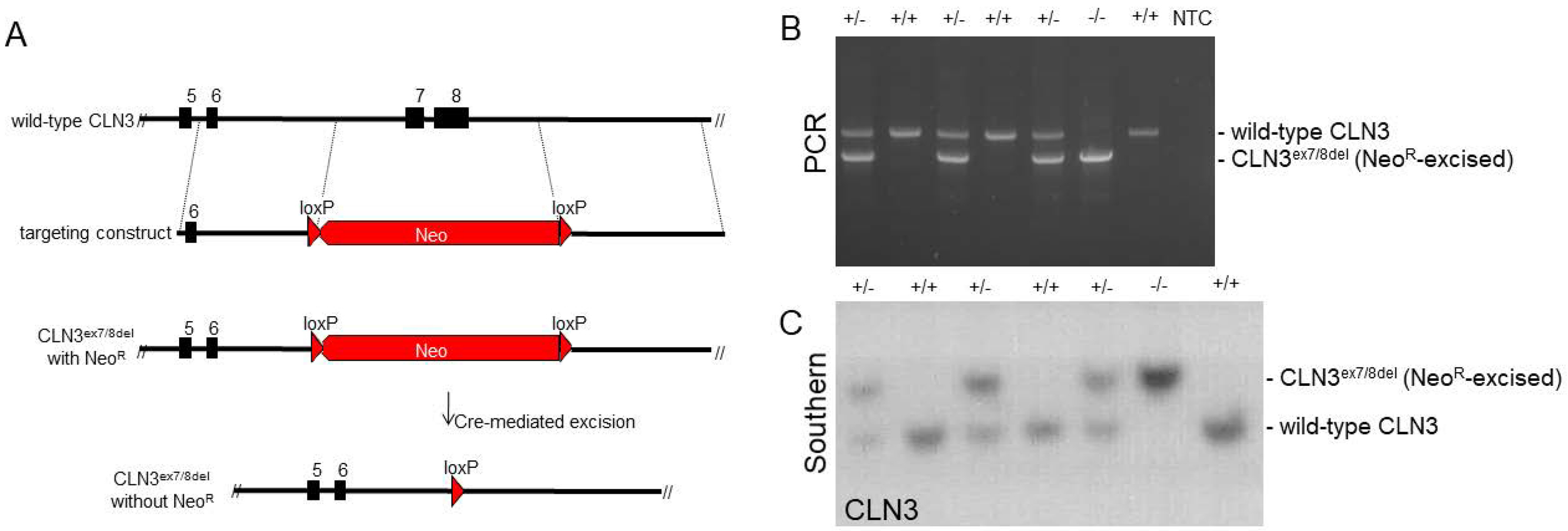

Fig. S1. ***CLN3$^{\Delta ex7/8}$* founder generation.** Schematic of gene targeting vector used to delete exons 7/8 from porcine *CLN3*. Porcine *CLN3* exons are depicted as black boxes. Neomycin resistance cassette (red) is driven by the phosphoglycerate kinase (PGK) promoter and flanked by loxP sites. Each homology arm is ~1.4 kb in length. Following Cre-mediated excision, a single loxP site remains (A). PCR confirmation of *CLN3$^{\Delta ex7/8}$* recombination in miniswine; WT: lanes 2, 4, 7; Heterozygote: 1, 3, 5; Homozygote: lane 6. Lane 8 is a no template control (B). Southern blot confirmation of *CLN3$^{\Delta ex7/8}$* allele in miniswine; WT: lanes 2, 4, 7; Heterozygote: 1, 3, 5; Homozygote: lane 6 (C). When probed for NeoR, no signal was detected, as would be expected following NeoR excision (data not shown).

Disease Models & Mechanisms • Supplementary information

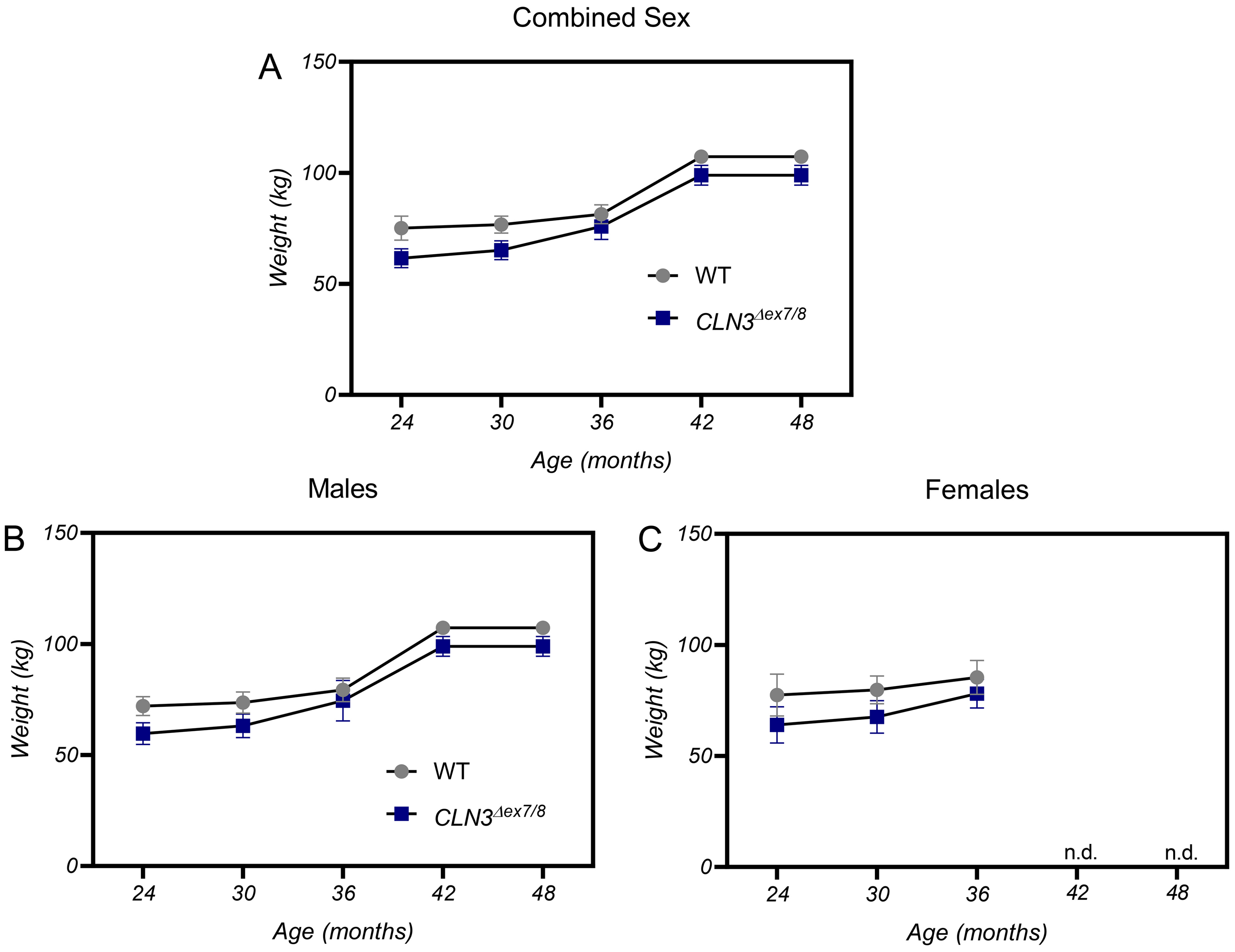

Fig. S2. **Weight of *CLN3*$^{\Delta ex7/8}$ and wild-type miniswine.** (A) Monthly weights for combined sex, no differences between genotypes (B). Monthly weights for males only. No significant differences are seen between *CLN3*$^{\Delta ex7/8}$ males and wild-type males (C). Monthly weights for females. *CLN3*$^{\Delta ex7/8}$ females weighed similar to wild-type females. Mean ± SEM. Mixed-model ANOVA with Sidak's multiple comparisons.

Disease Models & Mechanisms • Supplementary information

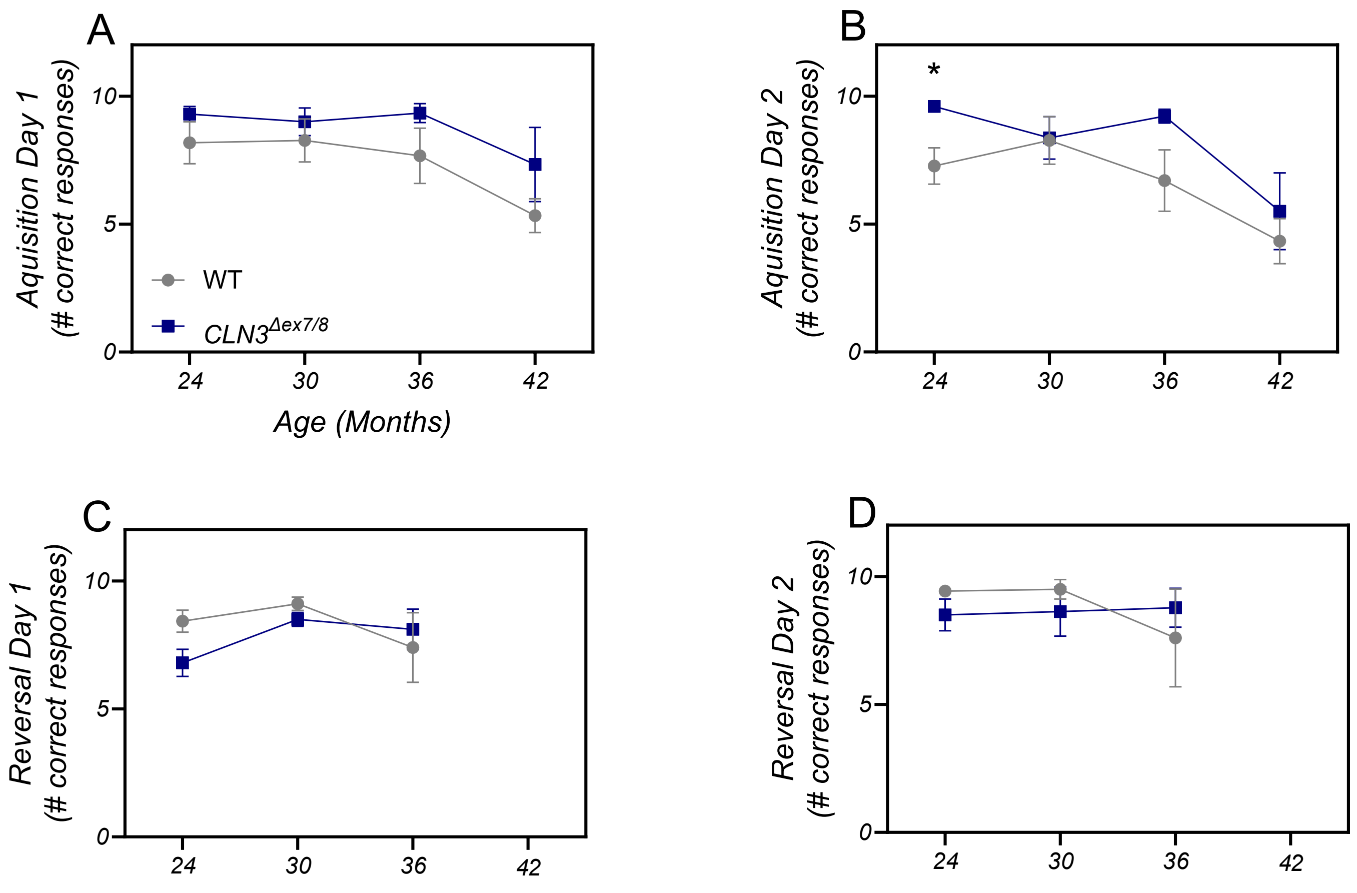

Fig. S3. ***CLN3$^{\Delta ex7/8}$* miniswine do not show learning deficits.** All animals perform similarly during Acquisition Day 1 (memory test) (A), Acquisition Day 2 (memory test) (B), and Reversal Day 1 (learning test) (C), with a similar number of correct choices. However, at 42 months of age, no animals were able to complete the learning test due to poor performance in the memory tests (<80% accuracy; n.d. represents no data). All animals perform similarly during Reversal Day 2 (learning test), with a similar number of correct choices (D). Mean ± SEM. Mixed-model ANOVA with Sidak's multiple comparisons. Animal numbers in Table S4.

Disease Models & Mechanisms • Supplementary information

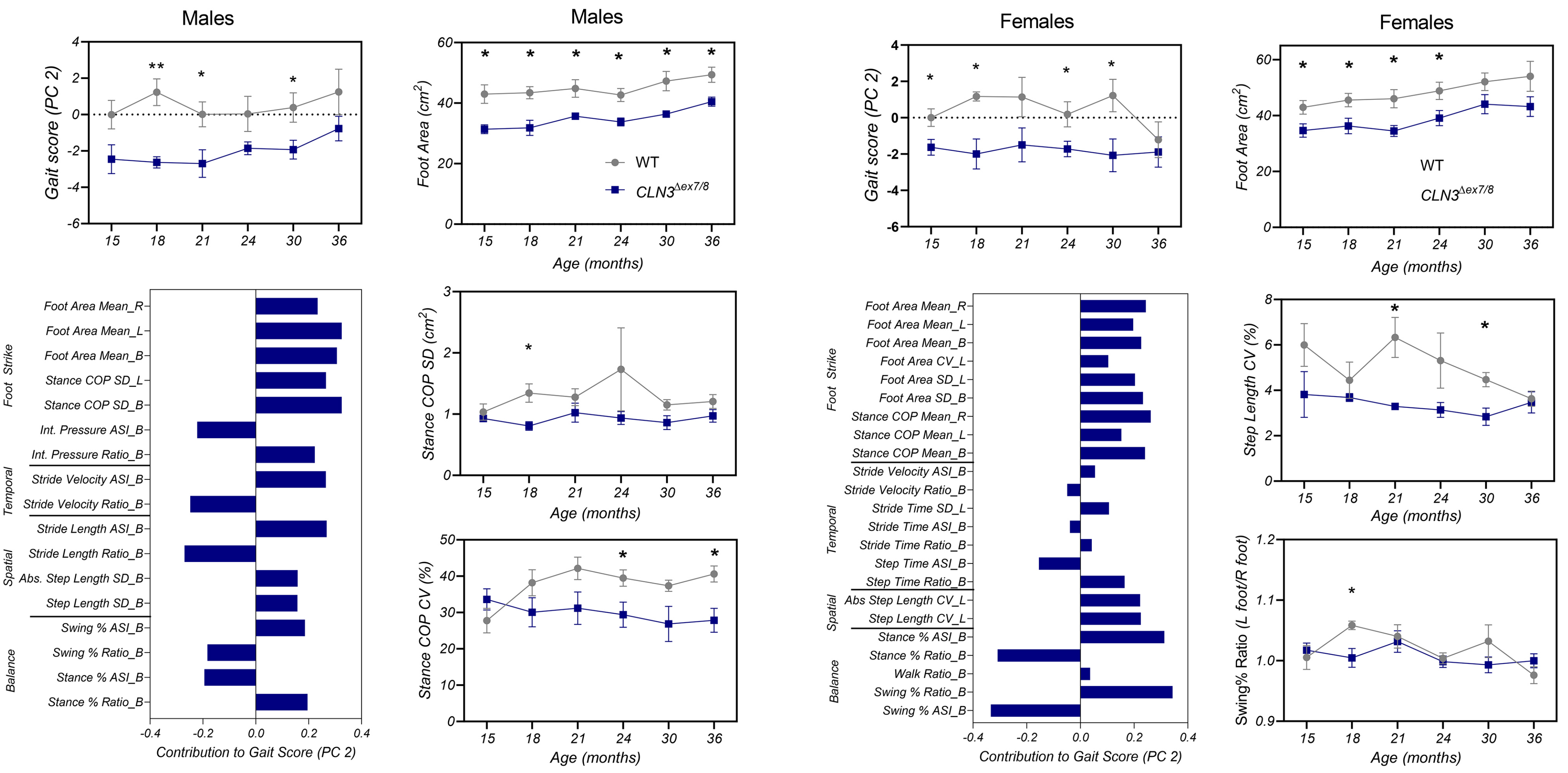

Fig. S4. *CLN3$^{\Delta ex7/8}$* animals have a significantly different front gait than wild-type animals. PCA gait score of male-only (A) and female-only (F) datasets from front feet. Description of contributing variable to gait scores from male-only (B) and female-only (G) datasets. Foot area for male-only (C) datasets. *CLN3$^{\Delta ex7/8}$* male miniswine have significantly smaller footfall sizes compare to wild-type at all time points. Stance Center of Pressure (COP) variables for male-only (D-E) datasets. *CLN3$^{\Delta ex7/8}$* male miniswine have shorter stance COP distance and less variability in stance COP trajectories, indicative of a cautious balancing strategy and more controlled stance. *CLN3$^{\Delta ex7/8}$* females show less variability in step length at 21 and 30 months of age and show less variability in swing% between right and left feet (J). Two-way ANOVA with uncorrected Fisher's LSD. *p≤0.05, **p≤0.01. B=both, R=right, L=left, CV=coefficient of variation. Animal numbers in Table S4.

Disease Models & Mechanisms • Supplementary information

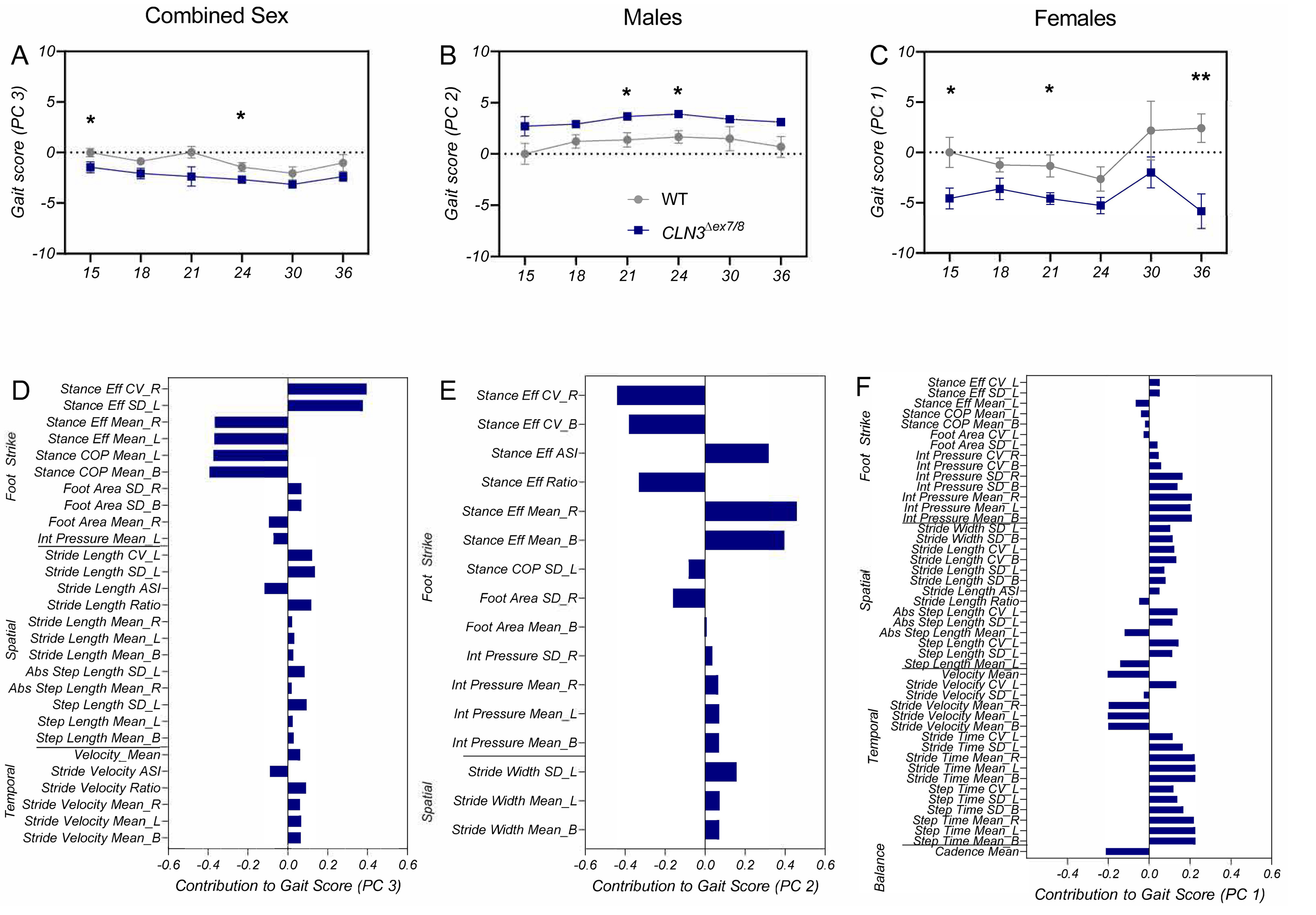

Fig. S5. CLN3 $^{\Delta ex7ia}$ animals have a significantly and subtly different hind gait than wild-type animals at 15 and 24 months of age. PCA gait score of combined sex (A), male-only (B), and female-only (C) datasets from hind feet. Description of contributing variable to gait scores from combined sex (D), male-only (E), and female-only (F) datasets. Mixed-model ANOVA with uncorrected Fisher's LSD. *p:50.05, **p:50.01.

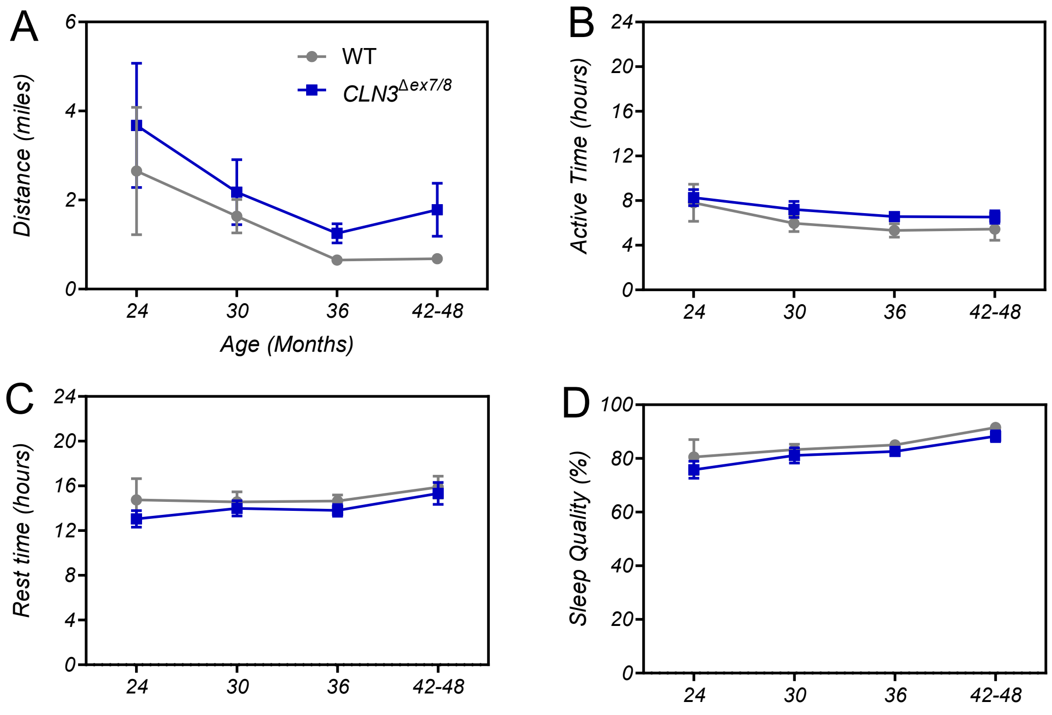

Fig. S6. **Comparison of** ***CLN3$^{\Delta ex7/8}$*** **and wild-type animals regarding distance traveled, active/rest time and sleep quality.** No significant differences were observed in home pen activity when using a Fitbark activity monitoring device, including total distance travelled (A), time active (B), time resting (C), and sleep quality (D). Mean ± SEM. Two-way ANOVA, Fisher's LSD. Animal numbers in Table S4.

Disease Models & Mechanisms • Supplementary information

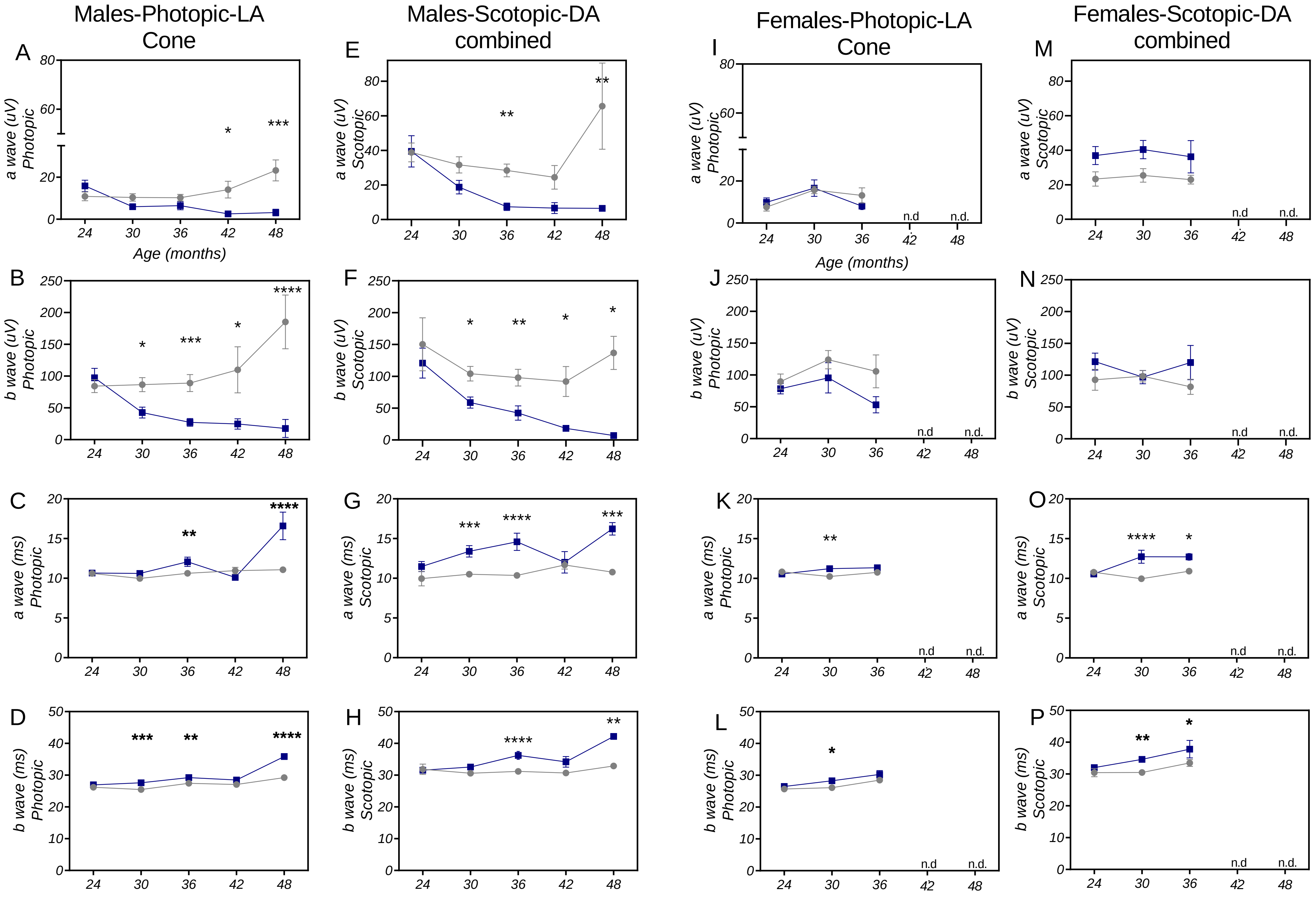

Fig. S7. *CLN3*$^{\Delta ex7/8}$ males show electroretinogram (ERG) amplitude and latency differences by 30 months of age, whereas *CLN3*$^{\Delta ex7/8}$ females only show latency differences. *CLN3*$^{\Delta ex7/8}$ males show photopic a-wave amplitude declines at 42 months (A) and b-wave amplitude declines at 30 months (B); Latency delays arise at 36 months in a-wave (C) and slightly earlier at 30 months in b-waves (D). *CLN3*$^{\Delta ex7/8}$ male miniswine show declines in scotopic a-wave (E) and b-wave (F) amplitudes by 36 and 30 months of age, respectively. Delayed scotopic a-wave latencies arise at 30 months of age in all *CLN3*$^{\Delta ex7/8}$ miniswine (males and females)(G,O). Delayed photopic and scotopic b-wave latencies arise at 30 months in *CLN3*$^{\Delta ex7/8}$ female miniswine (L, P), however scotopic b-wave latencies are slightly later in *CLN3*$^{\Delta ex7/8}$ miniswine males at 36 months (H). Mean± SEM. Two-way ANOVA, Fisher's LSD. *$p \leq 0.05$, **$p \leq 0.01$,***$p \leq 0.001$,****$p \leq 0.0001$. Photopic: 8.0cd s/m2 flash @2.0 Hz (cone predominant). Scotopic: 8.0 cd s/m2 flash @ 0.1 Hz (mixed rod and cone response). Absolute value of a-wave amplitude shown. Animal numbers in Table S4.

Disease Models & Mechanisms • Supplementary information

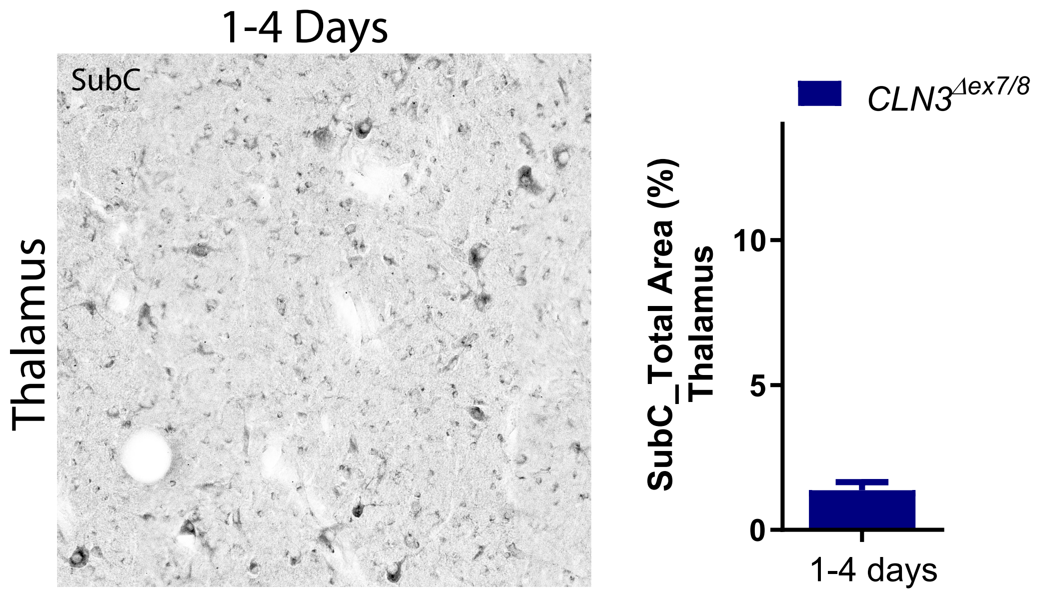

Fig. S8. Subunit C accumulation was evident at 1-4 days in the thalamus of *CLN3*$^{\Delta ex7/8}$ miniswine. Mean ± SEM.

Disease Models & Mechanisms • Supplementary information

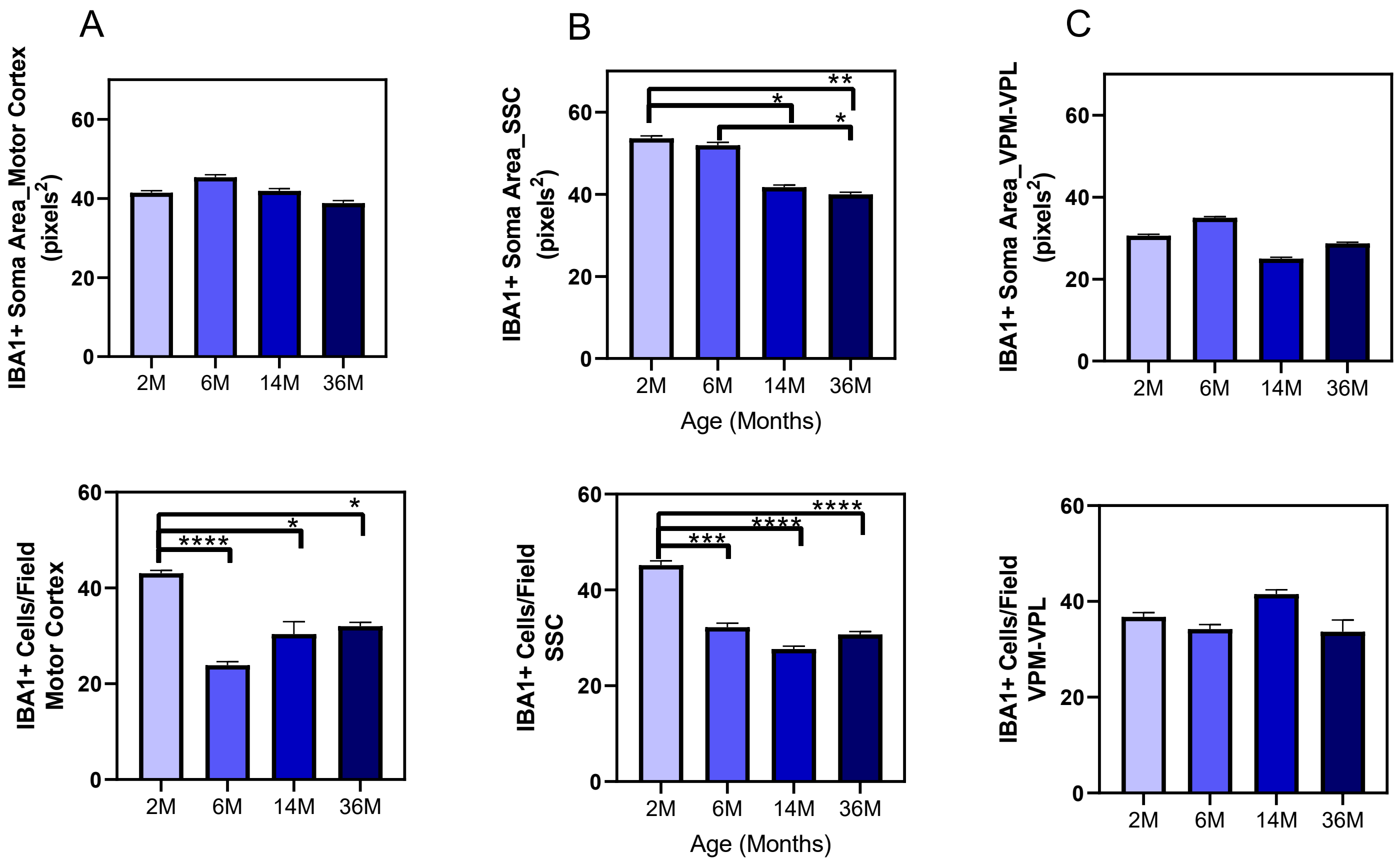

Fig. S9. IBA1+ soma sizes and number of IBA1+ cells in pooled genotypes (both WT and *CLN3*$^{\Delta ex7\text{-}8}$ miniswine) per timepoint. The sizes of the IBA1+ somas are smaller in older animals (14 and 36M) in the somatosensory cortex. More IBA+1 cells are found in 2-month old animals in both the motor and somatosensory cortex.

Disease Models & Mechanisms • Supplementary information

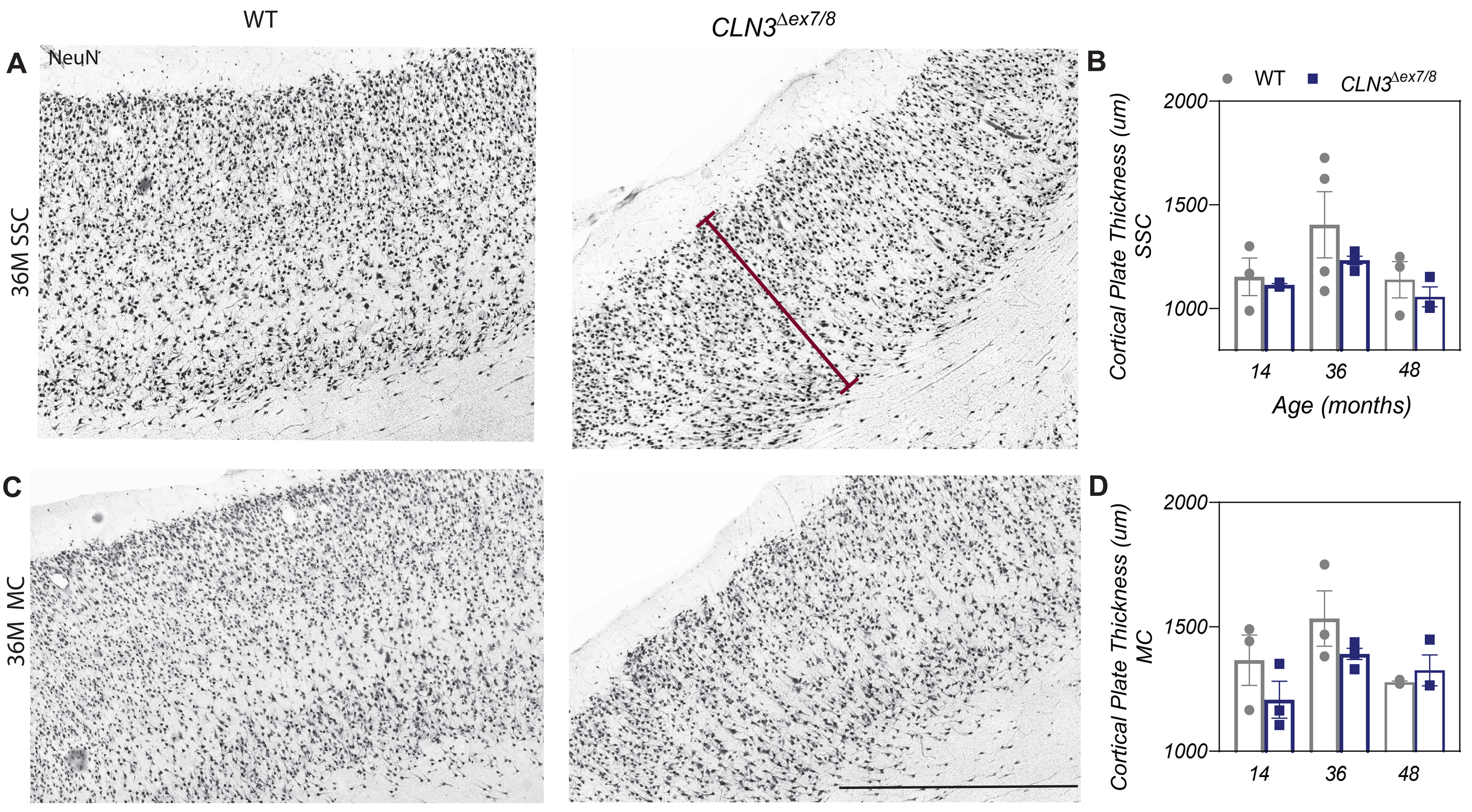

Fig. S10. ***CLN3***$^{\Delta ex7/8}$ **animals do not show cortical atrophy at 14, 36 or 48 months of age in either the somatosensory cortex (A-B) or motor cortex (C-D).** Neurons labeled with NeuN. Mean ± SEM. Unpaired t-tests. Scale bar=700 μm. Red bar indicates how cortical plate thickness was quantified. Animal numbers in Table S4.

Disease Models & Mechanisms • Supplementary information

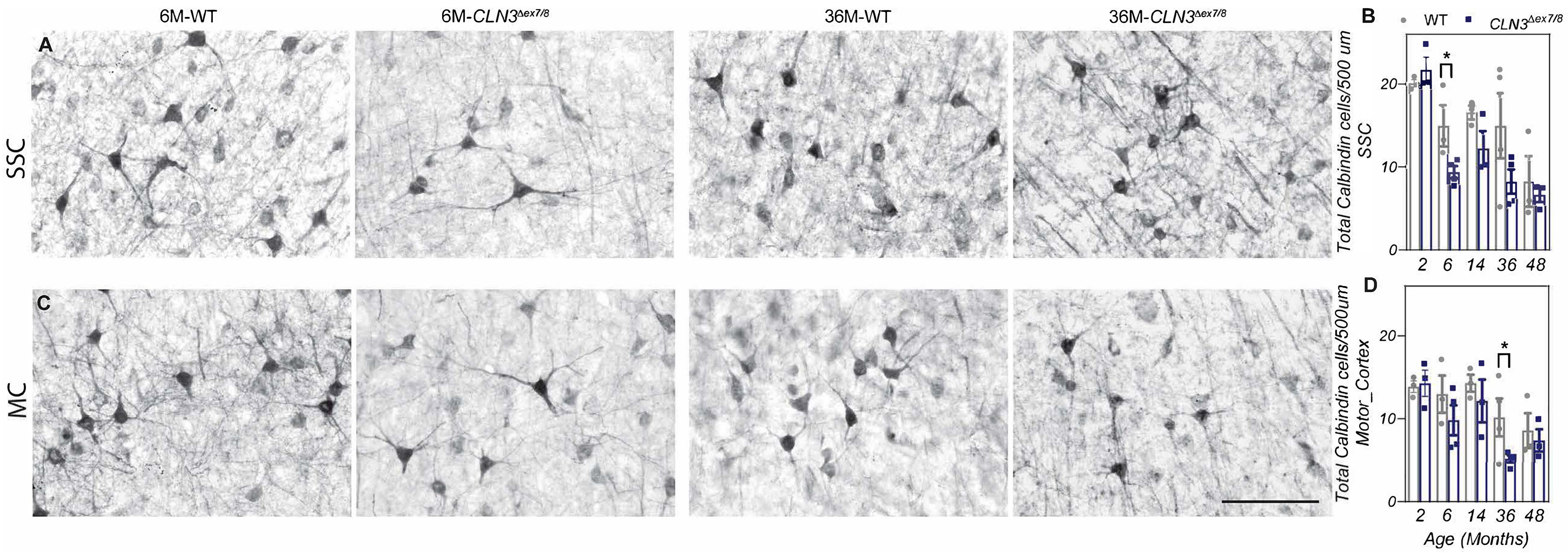

Fig. S11. From 6 to 36 months of age, a reduced number of calbindin+ interneurons are found in the somatosensory cortex (A-B) and motor cortex (C-0). Mean± SEM, unpaired t-test, *p=0.05, Scale bar=200 μm. Animal numbers in Table S4.

Table S1. Significant gait variables, time points in which the variables were significantly different between *CLN3*$^{\Delta ex7/8}$ and WT animals for combined sexes, and the % contribution of each variable to the PCA.

Combined Sex		
Gait variable	Time points	PC-% contibution
Step Time CV L	15M, 21M	2.87
Int Pressure SD R	15M, 24M	5.97
Foot Area SD B	15M, 18M	6.10
Stance COP Mean B	15M, 18M	6.50
Stance COP SD L	18M, 21M, 36M	8.77
Stance COP SD R	18M, 30M, 36M	10.25
Stance COP SD B	15M, 18M, 36M	12.20
Foot Area Mean R	15M, 18M, 21M, 24M, 30M, 36M	14.55
Foot Area Mean L	15M, 18M, 21M, 24M, 30M, 36M	15.82
Foot Area Mean B	15M, 18M, 21M, 24M, 30M, 36M	16.96

Male only		
Gait variable	Time points	Final PC-% contribution
Step Length SD B	N/A	2.48
Abs. Step Length SD B	N/A	2.51
Swing % Ratio	21M	3.34
Swing % ASI	21M	3.46
Stance % ASI	N/A	3.79
Stance % Ratio	N/A	3.83
Int. Pressure ASI	18M	4.91
Int. Pressure Ratio	18M	4.99
Foot Area Mean R	15M, 18M, 36M	5.48
Stride Velocity Ratio	18M	6.14
Stride Velocity ASI	18M	7.02
Stance COP SD L	36M	7.04
Stride Length ASI	N/A	7.19
Stride Length Ratio	N/A	7.26
Foot Area Mean B	15M, 18M, 21M, 24M, 30M, 36M	9.41
Stance COP SD B	18M	10.56
Foot Area Mean L	15M, 18M, 21M, 24M, 30M	10.58

Female only		
Gait variable	Time points	Final PC-% contribution
Walk Ratio	36M	0.13
Stride Time ASI	N/A	0.15
Stride Time Ratio	N/A	0.18
Stride Velocity Ratio	24M	0.24
Stride Velocity ASI	24M	0.29
Foot Area CV L	N/A	1.08
Stride Time SD L	N/A	1.13
Stance COP Mean L	N/A	2.32
Step Time ASI	N/A	2.38
Step Time Ratio	N/A	2.71
Foot Area Mean L	N/A	3.88
Foot Area SD L	N/A	4.13
Abs Step Length CV L	21M, 30M	4.95
Step Length CV L	21M, 30M	5.07
Foot Area Mean B	18M, 21M	5.12
Foot Area SD B	N/A	5.43
Stance COP Mean B	N/A	5.79
Foot Area Mean R	15M, 18M, 21M, 24M	5.93
Stance COP Mean R	21M	6.87
Stance% Ratio	18M	9.49
Stance % ASI	18M	9.78
Swing% ASI	18M	11.13
Swing% Ratio	18M	11.80

In males and females only, the variables that remained at the final PCA, time points in which those variables were significant, and contribution of each variable to the final PCA. SD-standard deviation, CV-coefficient of variation, R-right, L-left, B-both.

Table S2. Primers used for sequencing, targeting vector, rAAV production, and cell screening.

CLN3 primers for making targeting construct (5′ to 3′)	
pCLN3F3	GTT TAG CTG CTC TTA AAG GTA C
pCLN3R3	CTG CTG AGC ATG ACT TAG GA
pCLN3seqF9	TGA CTG CAC ACG TGG CAT GCA
pCLN3seqR10	GTG GCT CTG GTT CCC AGG TGC
pCLN3seqF12	TGG ACC CAG ACC CAA CAC CCA
pCLN3seqR15	TAG GGC AGC AGA TGG AGG CCA
pCLN3seqF14	TGC TCC TGG CAG ACA TCC TTC
pCLN3seqR13	AGC CGT GGA GAC AGA GTT ACA
pCLN3seqF11	GCT CCT GGG CCT CTG CAA CAA
pCLN3seqF19	GTG AGG AAG TGT CAT GGT CTG
pCLN3seqR20	CTA TTG GCA TCC AGC AGG TAG
pCLN3Ex6F7	AGC TTC ATC TTG GTC GCC TTC
pCLN3Ex7F4	TCT TGG CTA GCA TCT CTT CAG
pCLN3Ex8R4	GCT AGC ATC AGG GCA GGG ATA
pCLN3Ex9F17	TCC TGT TGC TCA CGT CTC CTG
pCLN3seqR16	CAC AAT GCA GTG AGA CTT CTT
pCLN3Ex9R18	CGC TAT TTA TCA GGG GCT GCC
pCLN3seqR24	CAG CTA CAG CTC CAA TTG GAC
pCLN3seqF21-2	AGG CTC TGA TAG GCC TGT TTG
pCLN3seqR25	GTA TAC ATG TAT GTG TAA CTG
pCLN3seqF28	TTA GGG CCA GGC CTT GTA GAA
pCLN3seqR27	TTG ACT CCT GGC TCA CAC CAA
CLN35′armR(EcoRV)2	ATC TGG GAT ATC TGT GGC TGT GGC GTA GGC CTG
CLN35′armF(XhoI)2.	TGT GAG CTC GAG TGT AGG CCA GTA GCT ACA GCT
CLN33′armF(HindIII)2	TGG AGC AAG CTT TTG TTG GCT GTG TTG TAT GGG
CLN33′armR(HindIII)2	AAG GGG AAG CTT CGT CTC CTA CCT GGC TTC AAC
AAVCLN3NeoRF(NotI)	CAC TAG TCG CGG CCG CTA CCA CTG AGC CGC AAT GGG A
AAVCLN3NeoRR2	TAG GTC GCA GCG GCC GCT CAG GTC CTG TGT TGT TGT GGC TG
AAVF1	CTC TAG CTA TAG TTC TAG TGG
AAVR2	GTG GTA TGG CTG ATT ATG ATC
Screen R (NeoR),	AAG ACA ATA GCA GGC AAC AAC
pCLN32PCRF1	CTT ACC CTT ACT CTG GGT CTG TAG
pCLN33PCRR18	TGG TCA GAG AGG TAA AGT AAC
pCLN3probeF2	TAA GAA GCC AAT GCT GGA GTT
pCLN3probeR3	AAG TAT TCG GCG TGG CCG TGA
NeoR-F	GCC ATT GAA CAA GAT GGA TTG
NeoR-R	CTC GTC AAG AAG GCG ATA GAA
pCLN32PCRF2	CTG ACT CTT AAT AAT GAA GGC TGC
pCLN32PCRR12	TAT GAT GGA ACA CGT AAT GCG AGA

Disease Models & Mechanisms • Supplementary information

Table S3. Gait variables and their definitions as provided by PKMAS Software (ver. 509C1, Protokinetics LLC, Havertown, PA).

	Parameters	Definition
Foot Strike	Integrated Pressure (pressure x s)	Area under the footfall pressure curve during the ground contact, also known as plantar pressure.
	Foot Area (cm^2)	Area of the ellipse that better adjusts to the footprint.
	Stance COP Dist. (cm)	It is the start to end distance of the COP trajectory for a single footfall. The distance between the coordinates of the COP at initial contact and the coordinates at foot off. In a perfectly efficient COP trajectory, the path would follow a straight line. The further the trajectory deviates from the straight line, the less efficient the trajectory is.
	Stance COP Path Eff. %	(Stance COP Distance/Stance COP path length)*100.
Spatial Parameters	Step Length (cm)	Distance between corresponding successive points on the heel of opposite feet measured parallel to the direction of progression for the ipsilateral stride of which it is the second part.
	Absolute Step Length (cm)	Distance between corresponding successive points on the heel of opposite feet.
	Stride Width (cm)	Distance between a line connecting the two ipsilateral foot heel contacts (the stride) and the contralateral foot heel contact between those events and is measured perpendicular to the stride.
	Stride Length (cm)	The distance from the heel of one foot to the following heel of the same.
Temporal Parameters	Step Time (s)	Time taken for one step and is measured from first contact of one foot to the first contact of following other foot.
	Stride Time (s) or Gait Cycle	Time from first contact of one foot, to the following first contact of the same foot. Gait Cycle consists of two phases, Stance and Swing.
	Stride Velocity (cm/s)	Ratio of Stride Length by the Stride Time.
	**Velocity (cm/s)-Only Mean**	Velocity is obtained after dividing the sum of all Stride Length measurements, by the sum of all Stride Time measurements, from both feet. This measurement is statistically different from the mean of the Stride Velocity. The computation for the Mean Stride Velocity applies equal statistical weight to each stride. Whereas the Velocity computation is weighted by the Stride Time.
Balance	Stance %GC	The period of time when the foot is in contact with the ground.
	Swing %GC	The period of time when the foot is not in contact with the ground. It is complimentary to the stance phase.
	**Walk Ratio (cm./(steps/min))-Only Mean**	Ratio between step length and cadence, not normalized by height. Index of the overall neuromotor gait control.
	**Cadence (steps/min)-Only Mean**	It is obtained after dividing the number of footfalls minus one by the ambulation time. The ambulation time is the time elapsed between first contact of the first and the last footfalls, expressed in seconds.
For each Parameter N=11	Mean	Mean Both, Mean Left, Mean Right, Mean Ratio, Mean ASI
	SD	SD Both, SD Left, SD Right
	%CV	%CV Both, %CV Left, %CV Right

These 16 parameters fall into 4 gait categories (Foot Strike, Spatial, Temporal, and Balance). All 16 parameters are summarized into means; whereas only 13 of these parameters have means, standard deviations and coefficients of variation for each foot (total of 11 statistical measures for each parameter). Hence, that is total of 13*11=143. Plus the means from Total velocity, walk ratio, and cadence (143+3=146 total variables for each PCA).

Disease Models & Mechanisms • Supplementary information

Table S4. Total numbers of animals aged between 15 and 48 months used for Fitbark, T-maze, Walkway analyses and ERG testing, and of animals aged between 2 and 48 months used for histopath assays.

Behavior	WT (in months)							
	15	18	21	24	30	36	42	48
Gait	10 (6F,4M)	12 (6F,6M)	11 (5F,6M)	11 (6F,5M)	12 (6F,6M)	11 (4F,7M)	1 (1M)	2 (2M)
ERG				4 (2F,2M)	13 (6F,7M)	12 (4F,8M)	2 (2M)	2 (2M)

	WT				
	24	30	36	42	48
Fitbark	2 (2F)	9 (4F,5M)	10 (4F,6M)	2 (2M)	1 (1M)
T-maze	12 (6F,6M)	11 (5F,6M)	10 (4F,6M)	3 (3M)	2 (2M)

Behavior	$CLN3^{\Delta ex7/8}$ (in months)							
	15	18	21	24	30	36	42	48
Gait	9 (5F,4M)	10 (6F,4M)	6 (3F,3M)	10 (5F,5M)	10 (5F,5M)	10 (5F,5M)	2 (1F,1M)	2 (2M)
ERG				8 (3F,5M)	12 (5F,7M)	11 (5F,6M)	3 (1F,2M)	3 (1F,2M)

	$CLN3^{\Delta ex7/8}$				
	24	30	36	42	48
Fitbark	5 (2F,3M)	9 (4F,5M)	11 (5F,6M)	2 (1F,1M)	2 (1F,1M)
T-maze	10 (5F,5M)	11 (5F,6M)	9 (5F,4M)	3 (1F,2M)	2 (2M)

Histopath	WT (in months)				
	2	6	14	36	48
SubC	3 (2F,1M)	3 (2F,1M)	4 (1F,3M)	4 (2F,2M)	3 (1F,2M)
GFAP	3 (2F,1M)	3 (2F,1M)	4 (1F,3M)	3(1F,2M)	3 (1F,2M)
IBA1	3 (2F,1M)	3 (1F,2M)	3 (1F,2M)	4 (2F,2M)	3 (1F,2M)
Calbindin	3 (2F,1M)	3 (1F,2M)	3 (1F,2M)	6 (2F,4M)	3 (1F,2M)
Cortical Plate			3 (1F,2M)	4 (2F,2M)	3 (1F,2M)

Histopath	$CLN3^{\Delta ex7/8}$ (in months)				
	2	6	14	36	48
SubC	3 (2F,1M)	4 (2F,2M)	3 (3M)	5 (3F,2M)	3 (1F,2M)
GFAP	3 (2F,1M)	4 (2F,2M)	3 (3M)	4 (3F,1M)	3 (1F,2M)
IBA1	3 (2F,1M)	4 (2F,2M)	3 (3M)	4 (2F,2M)	3 (1F,2M)
Calbindin	3 (2F,1M)	4 (2F,2M)	3 (3M)	4 (2F,2M)	3 (1F,2M)
Cortical Plate			3 (3M)	4 (2F,2M)	3 (1F,2M)
